# Insights From a Multi‐Method Recharge Estimation Comparison Study

**DOI:** 10.1111/gwat.12801

**Published:** 2018-07-19

**Authors:** David Walker, Geoff Parkin, Petra Schmitter, John Gowing, Seifu A. Tilahun, Alemseged T. Haile, Abdu Y. Yimam

**Affiliations:** ^1^ School of Engineering Newcastle University Newcastle upon Tyne, UK; ^2^ International Water Management Institute (IWMI) Addis Ababa Ethiopia; ^3^ School of Agriculture, Food and Rural Development Newcastle University Newcastle upon Tyne, UK; ^4^ Faculty of Civil and Water Resources Engineering, Bahir Dar Institute of Technology Bahir Dar University Bahir Dar, Ethiopia

## Abstract

Although most recharge estimation studies apply multiple methods to identify the possible range in recharge values, many do not distinguish clearly enough between inherent uncertainty of the methods and other factors affecting the results. We investigated the additional value that can be gained from multi‐method recharge studies through insights into hydrogeological understanding, in addition to characterizing uncertainty. Nine separate groundwater recharge estimation methods, with a total of 17 variations, were applied at a shallow aquifer in northwest Ethiopia in the context of the potential for shallow groundwater resource development. These gave a wide range of recharge values from 45 to 814 mm/a. Critical assessment indicated that the results depended on what the recharge represents (actual, potential, minimum recharge or change in aquifer storage), and spatial and temporal scales, as well as uncertainties from application of each method. Important insights into the hydrogeological system were gained from this detailed analysis, which also confirmed that the range of values for actual recharge was reduced to around 280‐430 mm/a. This study demonstrates that even when assumptions behind methods are violated, as they often are to some degree especially when data are limited, valuable insights into the hydrogeological system can be gained from application of multiple methods.

## Abstract 2

*Article impact statement:* Analysis of common recharge methods shows the value of extracting additional information to improve hydrogeological understanding.

## Introduction

Estimates of groundwater recharge allow quantification of renewable groundwater resources and can be used to indicate aquifer vulnerability to contamination or drought, assess groundwater contribution to streams (baseflow) and wetlands, and identify the implications of changes to land use, land cover or climate (Misstear 2000; de Vries and Simmers 2002; Healy 2010). Several notable reviews published over the past decades discuss various methodologies of estimating groundwater recharge ( Simmers 1988; Lerner et al. 1990; Scanlon et al. 2002; Healy 2010). It is well known, and stated by these reviews, that groundwater recharge estimates often vary between methods due to the uncertainties inherent with each method, the different spatiotemporal scales at which they operate, and the type of recharge they represent. It is normally recommended, therefore, that multiple methods are used. However, recharge estimation methods are often chosen in practice according to data availability even though the method may not be the most suitable for the particular climate or hydrogeological conceptual model. Often, the violation of a method's assumptions may only become apparent when the recharge result is compared to results from different methods. Also, some recharge estimation studies do not make a clear distinction between the reasons why the recharge results differ, whether it is due to genuine uncertainties in data and methods, unsatisfied assumptions, different spatiotemporal scales, or if the method is actually computing a different type of recharge. However, recognizing these distinctions in multi‐method recharge estimation comparison studies can help to provide useful insights into the hydrogeological system.

A recharge assessment was conducted at a study site in northwest Ethiopia (Dangila *woreda*, a local administrative district), in the context of an investigation into the resilience of shallow groundwater resources used for irrigation by rural communities. Following recommended approaches, for example, Scanlon et al. (2002) and Healy (2010), several techniques were initially applied, and it was found that they gave a wide range of recharge estimates. This is commonly reported in the literature, for example, Berehanu et al. (2017); Afrifa et al. (2017), although it is less common for studies to report investigation of the reasons for the range of values. Some previous studies, typically using at most three to five recharge estimation techniques, have considered the basis for differing recharge estimates in more detail, and concluded that the range of recharge estimates contains useful information to inform further understanding of the conceptual model (e.g., Coes et al. 2007; King et al. 2017). For our study, there were sufficient data of suitable quality to apply a larger number of recharge estimation methodologies at a single site, so a wider investigation was made to assess which of the most commonly applied recharge estimation methods could help to provide insights and increase understanding of the hydrogeological system. Nine different recharge estimation techniques were applied, with a total of 17 variations, including variants of methods and variations in how input data were derived. The methods are presented here in order of increasing data requirement and complexity: an empirical method, streamflow hydrograph methods (three variations), soil moisture balance (two variations), basin water balance (three variations), chloride mass balance, water table fluctuation (two variations), rainfall infiltration breakthrough, and physically‐based modeling. The ninth method is large‐scale mapping and modeling (three variations) from which recharge values have been obtained for comparison from published studies.

The three aims of this paper are to:

1. Demonstrate quantitatively the range of recharge results that can be calculated from as many methods as feasible for the study site, and analyze the underlying reasons for the different recharge values
2. Assess the utility of applying multiple methods in order to gain insights on the hydrogeological system
3. Provide a recharge estimate with uncertainty for Dangila *woreda*.

The study highlights and analyses the general problem of interpretation of variability in recharge estimates obtained from different methods. It is noted that all methods were applied even if assumptions may not be fully complied with, since this is a factor relevant to uncertainty in recharge estimation in many published studies. It is not uncommon for recharge results to be reported without explicit statement of assumptions and limitations or the type of recharge being computed (Sophocleous 1985; Wood 1999; Halford and Mayer 2000). It may only be through identifying significant discrepancies between recharge results from different methods that violation of a method's assumptions are realized and the hydrogeological conceptual model can be amended and better understood. In addition, this study provides a useful recharge estimate for a shallow aquifer in northwest Ethiopia. Published recharge estimation studies from sub‐Saharan Africa are not great in number, not well geographically distributed, and many are gray literature (Bonsor and MacDonald 2010; Wang et al. 2010; Pavelic et al. 2012 and Chung et al. 2016). The majority of studies are concentrated in arid and semi‐arid regions due to water scarcity in these areas. However, many regions of apparent high rainfall also experience water scarcity during the dry season (Rijsberman 2006) and when sub‐Saharan Africa's variable climate unpredictably delivers low‐wet season rains (Van Koppen 2003; Bonsor et al. 2010).

### Groundwater Recharge

Lerner et al. (1990) provide the classical definition of recharge: “the downward flow of water reaching the water table, forming an addition to the groundwater reservoir.” This defines “actual recharge” and is referred to as such by many authors, for example, Scanlon et al. (2002), Healy (2010), Misstear et al. (2007). According to Rushton (1997), the term “actual recharge” is used to distinguish it from *potential* or *minimum* recharge. *Potential* recharge is water passing downward through the unsaturated zone that could potentially contribute to the aquifer. *Potential* recharge is the term used by many authors for recharge computed from unsaturated zone methods as this infiltrated water may be subject to losses (e.g., root zone uptake, interflow then surface discharge) before contributing to the aquifer (e.g., Simmers 1988; Rushton 1997; Healy 2010). *Minimum* recharge refers to groundwater discharge to rivers or springs, when the two quantities are considered to be in balance. It is termed *minimum* recharge because other losses (e.g., evaporation from the saturated zone, seepage to deeper aquifers) may have occurred since the water was recharged (e.g., Szilagyi et al. 2003; Vegter et al. 2003; Risser et al. 2005).

In humid regions characterized by shallow water tables and gaining rivers, diffuse (or direct) recharge dominates. In arid regions characterized by deep water tables and losing rivers, recharge is usually focussed (or indirect) along river corridors with rates generally limited by water availability at the surface (Allison 1988; Scanlon et al. 2002). The factors that influence the amount and type of recharge (diffuse or focussed) include: precipitation (volume, intensity, and duration); topography (slope, above ground storage); vegetation (cropping pattern, rooting depth) and evapotranspiration; soil and subsoil types; flow mechanisms in the unsaturated zone (uniform or preferential); bedrock geology; available groundwater storage; presence of influent rivers, and; presence of karst features (Misstear 2000).

### Recharge Estimation Methods

Various techniques are available for estimating recharge, the selection of which is not straightforward (Lerner et al. 1990; Scanlon et al. 2002). Each technique has different assumptions as well as limitations. Therefore, it is recommended to use multiple methods to reduce uncertainty and to improve conceptual understanding of recharge at a study site (de Vries and Simmers 2002; Healy and Cook 2002). Generally, selection of a technique is dependent on data availability, which is often lacking in many regions. Such data scarcity can lead to the selection of a less suitable recharge estimation method as well as no additional methods to corroborate the findings. Rather than data driving the methodology used, the user should select methodologies depending on the desired spatiotemporal resolution. This is easier for primary data collection but less obvious when dependent on secondary data sources. Then the user must determine what the recharge result represents, according to the fundamental theory of the method applied and the satisfaction of the assumptions.

## Study Area

### General Description

**Figure 1:**
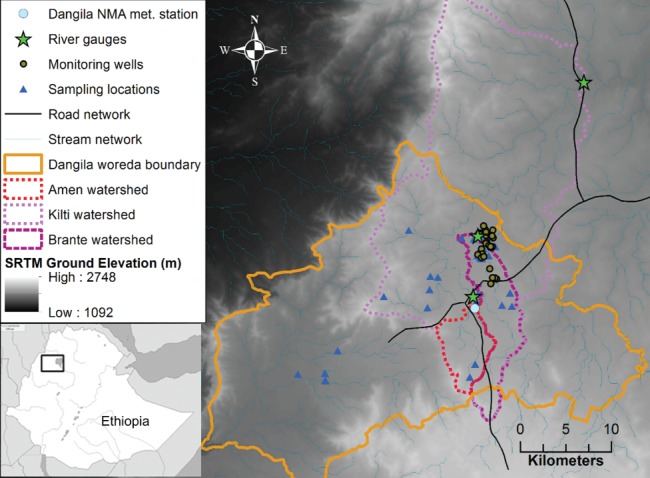
Location map of the study area.

The study site is Dangila *woreda* within the Amhara Region of northwest Ethiopia, 70 km southwest of Bahir Dar on the Addis Ababa to Bahir Dar road (Figure 1). The *woreda* (district) has an area of approximately 900 km^2^ and a population of around 175,000 of which 135,000 are rural (CSA 2012).

Elevation ranges from 1600 to 2400 m; the west drains to the Beles river, a tributary of the Blue Nile (Abay), while the east drains via the Gilgel Abay river into Lake Tana. Much of the district has low relief with expansive floodplains providing year‐round pasture and dwellings and crops occupying adjacent slopes. Cultivated land occupies 72% of the district where rainfed agriculture predominates, the main crops being *tef*, maize, barley, and millet (Belay and Bewket 2013; ADSWE 2015).

### Climate

**Figure 2:**
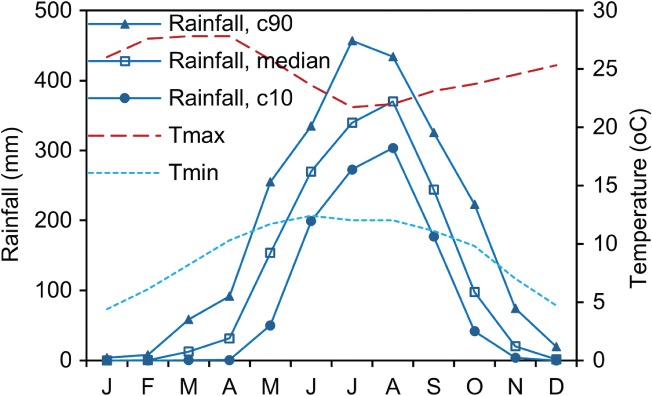
Monthly median, 10th and 90th percentile rainfall, and mean maximum and minimum temperatures as measured (1987‐2017) by the NMA at the Dangila weather station.

The climate of the region is moist subtropical with little annual temperature variation though high‐diurnal variation. The median annual total rainfall is 1541 mm, as measured (1987‐2017) at the National Meteorological Agency (NMA) weather station in Dangila town, 91% of which falls during May to October (Figure 2). Both the mountains to the east and Lake Tana to the north affect the pattern of rainfall in the study area. Most rain events have a duration shorter than 1‐h and often occur in the late afternoon (Haile et al. 2009).

### Hydrogeology

Bedrock geology consists of Cenozoic basalt and trachyte (Tefera et al. 1996) that are variously massive, fractured and vesicular. Above the bedrock lies weathered basalt regolith, itself overlain by red clayey loam soils (nitisol). The superficial materials of the floodplains are occasionally very sandy and gravelly though deep and wide desiccation cracks suggest a high‐clay content (vertisol). Local communities report that there are rarely problems with well sidewall collapse and the solid bedrock geology is often reached abruptly then well excavation is halted. Therefore, the location of the rockhead can be inferred from well depth and is generally found to be deeper (12‐15 m) in more steeply sloping areas and shallower in floodplains where wells are as little as 3 m in depth. The wet season water table approaches ground level in and around the seasonally inundated floodplains while on slopes and in hilly areas it rises to within 3‐4 m of ground level. Wells often dry out in the dry season.

**Figure 3:**
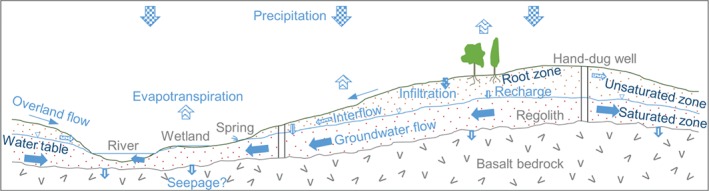
Conceptual model of the study site.

Diffuse (direct) recharge dominates across the study site (Figure 3) with quantities likely to vary according to local position. Upslope areas will receive less recharge due to higher runoff and interflow gradients whereas overland flow, interflow and groundwater flow collect in the topographic lows. The large floodplains, which are prevalent in the landscape, become waterlogged in the wet season from direct rainfall and spring discharge (rather than from overbank flooding).

### Data Used in the Study

In this study, nine frequently used methods were applied using data from all possible hydrological zones. Additional methods were explored and rejected for various reasons, as discussed in the online Appendix S1, Supporting Information. The data requirements for the various methods applied are shown in Table 1. Meteorological data was measured by the NMA weather station in Dangila town: the only formal weather station in the district. River flow data in Ethiopia is collected by the Ministry of Water, Irrigation, and Electricity: Amen and Kilti river flow data were utilized, the latter catchment forming a large portion of Dangila district, even though the gauging station lies outside the district (Figure 1). The available time series date from 1988 (Amen) and 1997 (Kilti) to late 2014, though there are occasional gaps in the data. In addition to these formal data sources, hydrometeorological time series are available from a community‐based monitoring program at Dangesheta village from March 2014 to January 2017. River stage in the Brante river was measured twice‐daily, rainfall was measured daily in a manual raingauge, and groundwater levels were measured bi‐daily in five wells since March 2014 and daily in 25 wells since February 2015. The hand‐dug wells have an average diameter of 1 m with depths ranging from 3 to 21 m. Rainfall and river stage from the community‐based monitoring have been validated against formal sources confirming the quality of the data (Walker et al. 2016). The Amen (37.0 km^2^) and Brante (65.5 km^2^) are sub‐catchments of the Kilti (631.7 km^2^). The catchment‐scale recharge assessment methods were applied to all three catchments. Thirty‐one shallow groundwater samples were collected for chloride analysis, from locations distributed throughout the study site, in March/April 2015 and October/November 2015. Rain could only be sampled during the second field visit because it did not rain during the 4 weeks of the earlier dry season visit, nor during a third visit in January 2017. Three samples were collected from two sites and occurred whenever rainfall was sufficient to enable direct sampling. All samples were filtered upon collection and, to prevent evaporation, the nalgene bottles were completely filled and kept in a refrigerator prior to laboratory analysis by Dionex ion chromatography. Additional data used in development of the conceptual model and required to parameterize models resulted from three periods of fieldwork, which included pumping tests on hand‐dug wells (Walker 2016), geological surveys, hydrochemistry and stable isotope sampling, radon‐222 measurements, water point surveys, and workshops with the local community (further information is provided in the Appendix S1). Proportions of different land use land cover (LULC) types were taken from ADSWE (2015).

**Table 1:** Hydrological Zone, Spatial Scale, and Data Requirements of the Applied Recharge Estimation Techniques

	Hydrological Zone	Spatial Scale of the Method	MeteoroLogical Averages	MeteoroLogical Time Series	River Flow Time Series	GroundWater Sampling	Rainfall Sampling	GroundWater Level Time Series	Aquifer Properties	Soil Properties	Vegetation Properties	Geological Surveys or Maps	Land Use/Land Cover Surveys or Maps	Digital Dlevation Model (DEM)	Access to Literature
Empirical	A	D	√												√^1^
Stream hydrograph	SW	C			√										
SMB	UZ	D		√											
Water balance	A	C		√	√										
CMB	SZ	D	√			√	√								
WTF	SZ	L						√	√						
RIB	SZ	L		√				√	√						
Physically‐based modeling	A	C		√	√			√	√	√	√	√	√	√	
Large‐scale mapping	A	R													√^2^

A = all zones; SW = surface water; UZ = unsaturated zone; SZ = saturated zone; R = regional (1000s km^2^); D = district (*woreda*); C = catchment (10s‐100s km^2^); L = local (100s m^2^).

1 Access to literature only required if developing a new empirical equation.

2 Assuming consideration of published studies as opposed to developing new large‐scale maps.

Data from three large‐scale mapping and modeling recharge studies were also assessed. The global‐scale WHYMAP (WHYMAP 2016) by BGR (the German Federal Institute for Geosciences and Natural Resources) and UNESCO gave recharge values of 20‐100 mm/a for the study site, the continental‐scale map by Altchenko and Villholth (2015) gave 100‐300 mm/a, and a national‐scale map by Ayenew et al. (2008) gave 250‐400 mm/a. Further information on the large‐scale mapping and modeling can be found in the Appendix S1.

## Recharge Estimation Methodologies

### Empirical Method

**Figure 4:**
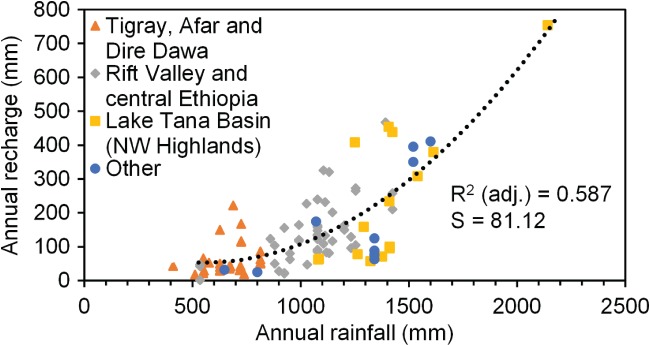
Plot showing the relationship between annual rainfall and annual recharge in Ethiopia based on 102 recharge estimates from 49 studies across the country. *S* = standard error, *R* ^*2*^ *(adj.)* = adjusted coefficient of determination. The Tigray, Afar, Dire Dawa group has semi‐arid climate and highly heterogeneous geology ranging from Precambrian crystalline to Mesozoic sandstones and limestones to Quaternary volcanics, generally overlain by leptosols with sparse and herbaceous vegetation. Rift Valley and central Ethiopia have subtropical highland and tropical savanna climate with Quaternary volcanic geology, highly heterogeneous soils and rainfed cropland and mosaic forest and grassland. The Lake Tana Basin has a tropical highland monsoon climate and Cenozoic volcanic rocks overlain by luvisols or vertisols closer to the lake with mosaic cropland/grassland/shrubland/forest (Tefera et al. 1996; Peel et al. 2007; Arino et al. 2012; Jones et al. 2013).

In an attempt to establish a rainfall‐recharge relationship for Ethiopia, a thorough and systematic literature search was conducted. The Appendix S1 provides detailed information on the literature search and a map of the study site locations, which were distributed around Ethiopia (Figure S1 in Appendix S1). Forty‐nine quantitative studies were located that provided 102 annual recharge estimates to plot against annual rainfall (Figure 4). A quadratic trendline, reflecting an increase in recharge disproportionate to increasing precipitation, achieved the best R^2^ and standard error. The resulting relationship is presented as Equation 1. Separating the data into the geographic (and consequently, climatic and geological) regions as shown in Figure 4 and fitting linear trendlines gave similar recharge values as the trendlines plot close to the quadratic regression line. Additional analysis of site‐specific, rather than regional, rainfall intensity, topography, soils and vegetation is beyond the scope of this study. The regression line is not extended to rainfall below 500 mm/a as this is considered the lower limit of applicability of Equation 1. Where rainfall is below 500 mm/a, the relationship with recharge is more complex (Bonsor and MacDonald 2010) and there were insufficient studies from which a relationship could be established.

(1) $$R=136.6-0.3005P+0.000271P^{2}$$

where *R* is recharge and *P* is annual precipitation.

### Streamflow Hydrograph Analysis (Three Methods)

Recharge estimation using streamflow hydrograph methods typically involves separating the baseflow component (Figure S2 in Appendix S1) and idealizing that precipitation entering the aquifer as recharge must be balanced by groundwater discharge into rivers that forms baseflow. However, there are several ways in which groundwater can be depleted without contributing toward baseflow, including abstractions, leakage to deeper aquifers, and evapotranspiration from the saturated zone. Without quantifying these fluxes, equating baseflow to recharge will lead to underestimation of recharge. It is important to remind, therefore, that quantifying baseflow is an estimate of groundwater discharge and provides a *minimum* estimate of recharge (Szilagyi et al. 2003; Vegter et al. 2003; Risser et al. 2005). Three streamflow hydrograph methods were used in this study, the baseflow recession method presented by Meyboom (1961), and two digital recursive filter tools, the Web GIS based Hydrograph Analysis Tool (WHAT) (Lim et al. 2005) and WETSPRO (Willems 2009). Details of the application of these methods are presented in the Appendix S1.

### Soil Moisture Balance (SMB)

The Thornthwaite and Mather (1955, 1957) (T‐M) method is essentially a water balance of the root zone performing monthly book‐keeping of precipitation, evapotranspiration and soil moisture. Deep infiltration below the root zone occurs only when field capacity is exceeded (Steenhuis and Van Der Molen 1986). The direct runoff component is dealt with by applying a runoff factor or by subtracting a portion of soil moisture surplus; both methods were applied here. Details of the parameterization and the tabulated calculations can be found in the Appendix S1.

A key assumption of unsaturated zone methods, such as the SMB method, is that the soil moisture surplus will infiltrate to the water table. However, this water may flow laterally through the unsaturated zone as interflow without necessarily recharging the aquifer (Misstear 2000; Hendrickx and Flury 2001). Hence, Simmers (1988), Rushton (1997), Healy (2010) and others refer to the recharge computed from unsaturated zone methods as *potential* recharge.

### Basin Water Balance

The water balance, or water budget, simplifies the full water balance equation by neglecting *Q*_*in*_, *A*, *Q*_*out*_ and *ΔS* in

(2) $$P+Q_{\text{in}}=\mathrm{RO}+\mathrm{AET}+R+A+Q_{\text{out}}+\Delta S$$

where *P* is precipitation, *Q*_*in*_ is groundwater flow into the basin, *RO* is runoff (i.e., overland flow and interflow out of the basin), AET is actual evapotranspiration (from the unsaturated and saturated zones and from surface water), *R* is recharge, *A* is abstraction, *Q*_*out*_ is groundwater flow out of the basin, and *ΔS* is the change in storage. The assumptions are that *ΔS* is balanced over long time‐periods (this appears valid from groundwater level records), *Q*_*in*_ and *Q*_*out*_ are negligible as these are headwater catchments with thin aquifers and rivers founded on bedrock (hence no groundwater flow beneath the gauge), and abstraction is negligible due to sparse wells with manual‐lifting technology. AET is not straightforward to estimate and was calculated with three methods for comparison: (1) The T‐M method; (2) Application of Turc's formula (Turc 1954), and; (3) A value estimated by Allam et al. (2016) for this region of the Tana Basin by combining remote sensing and river flow records. The average AET values were 789, 831 and 931 mm/a, respectively. See the Appendix S1 for details of the AET and runoff estimations. Accurate quantification of all the fluxes is always troublesome though is required in order to leave an accurate residual that is equated to *actual* recharge (Scanlon et al. 2002).

### Chloride Mass Balance (CMB)

The CMB method requires mean annual precipitation, chloride concentration of that precipitation and chloride concentration of the groundwater, is independent of whether recharge is diffuse or focussed, and integrates recharge rates both spatially across a region and temporally over long time‐periods. The method has several assumptions (Bazuhair and Wood 1996):

- All chloride within groundwater originates from precipitation, that is, there are no alternative chloride sources such as evaporites or pollution.
- Chloride is conservative in the system (this is generally the case as chloride is not adsorbed, is unlikely to form salts, and has rare biochemical interaction).
- Recycling of chloride does not occur within the basin area.
- The chloride concentration in runoff is equal to that in precipitation.
- Evaporation of groundwater does not occur upgradient of groundwater sampling points.

The basic equation applicable for evaluation of recharge using the CMB is

(3) $$R=\frac{\left(P_{\mathrm{eff}}\right)\left({\mathrm{Cl}}_{\mathrm{wap}}\right)}{{\mathrm{Cl}}_{\mathrm{gw}}}$$

where *R* is annual recharge, *P*_*eff*_ is average annual effective precipitation (rainfall minus direct runoff), *Cl*_*wap*_ is the weight‐average chloride concentration in precipitation including dry deposition, and *Cl*_*gw*_ is the average chloride concentration in groundwater. *Cl*_*gw*_ averaged 2.10 mg/L with a standard deviation of 1.33 mg/L and *Cl*_*wap*_ averaged 0.68 mg/L (standard deviation = 0.32 mg/L). Details of the parameterisation can be found in the Appendix S1.

### Water Table Fluctuation (WTF)

In the WTF method, the upward movement of groundwater level with respect to time is an indication of recharge and the downward movement indicates recession of groundwater; no assumptions are made regarding recharge mechanism (Healy and Cook [2002] for details). Groundwater recharge *R* is calculated for a particular well by multiplying the change in water level of two successive groundwater level readings by the specific yield *S*_*y*_ of the aquifer:

(4) R=Sy*ΔhΔt

where *h* is water level and *t* is time. To correctly estimate *Δh*, it is necessary to extrapolate the antecedent recession curve to the point below the peak, that is, the point that the groundwater level curve would have reached without precipitation (Figure S5 in Appendix S1). This extrapolation was conducted manually on each of the 30 well hydrographs, following the graphical method described by Delin et al. (2006). For comparison, another approach was followed that involves calculating the water level rise from 1 day to the next with a negative rise, that is, a fall in groundwater level, counting as zero. This method would be expected to underestimate recharge because groundwater recession with the absence of recharge is not considered (e.g., Delin et al. 2006, Varni et al. 2013, Choi et al. 2007). *S*_*y*_ of 0.08 was used, obtained from 11 pumping and recovery tests in the area (Walker 2016).

### Rainfall Infiltration Breakthrough (RIB)

The RIB method is a model for groundwater recharge estimation developed by Xu and Beekman (2003) based on the cumulative rainfall departure method (Wenzel 1936). The conditions at the field site fit well the requirements detailed by Sun et al. (2013): “… the RIB model is best suited for shallow unconfined aquifers with relatively low transmissivity.” The model considers not only rainfall from a single event but the series of preceding events that influence breakthrough of water at the water table (for details, see Xu and van Tonder 2001; Sun et al. 2013). Time series of rainfall are required, plus groundwater level and aquifer *S*_*y*_. The RIB method utilized data from the 30 community‐monitored wells and raingauge in addition to *S*_*y*_ of 0.08 (Walker 2016). Further details can be found in the Appendix S1. As with the WTF method, there is the possibility of accounting for groundwater level rise from lateral flows in recharge estimation.

### Physically‐Based Modeling

Système Hydrologique Européen TRANsport (SHETRAN) is a physically‐based spatially distributed finite difference modeling system for coupled surface and subsurface water flow in river basins and is openly available at http://research.ncl.ac.uk/shetran. SHETRAN is well established in the literature, having been applied to a variety of situations (e.g., Birkinshaw and Ewen 2000; Bathurst et al. 2011; Starkey et al. 2017), however, it has not previously been used to quantify recharge. Model setup requires a DEM, catchment mask, geological, soil, vegetation and LULC information. Further details of SHETRAN, including how recharge is computed within the model and how the models were parameterized, can be found in the Appendix S1. Three nested catchments were modeled, details of which are in Table 2. The calibration procedure involved adjusting geological layer thicknesses, aquifer properties, channel characteristics, Strickler overland flow roughness coefficient, and evapotranspiration characteristics until satisfactory matches with observed groundwater level and river discharge data were achieved. The nested nature of the catchments meant a final matching set of optimum parameters was selected to satisfy the calibration requirements of all catchments. Table 2 shows calibration statistics for a calibration period; subsequent simulations during a validation period were deemed acceptable (Appendix S1).

**Table 2:** Details of the Three Catchments Modeled using SHETRAN (Figure 1 for Locations)

Catchment	Area (km^2^)	Resolution (m)	Run Length	Calibration	NSE	RMSE
Amen	37	100 × 100	17 years (January 98 to September 14)	River flow	0.79	0.19 m^3^/s
Kilti	632	500 × 500	18 years (April 97 to October 14)	River flow	0.78	1.47 m^3^/s
Brante	66	100 × 100	3 years (March 14 to January 17)	GW levels	0.69	2.01 m

*NSE* = Nash‐Sutcliffe efficiency; *RMSE* = root mean square error.

## Recharge Results

**Figure 5:**
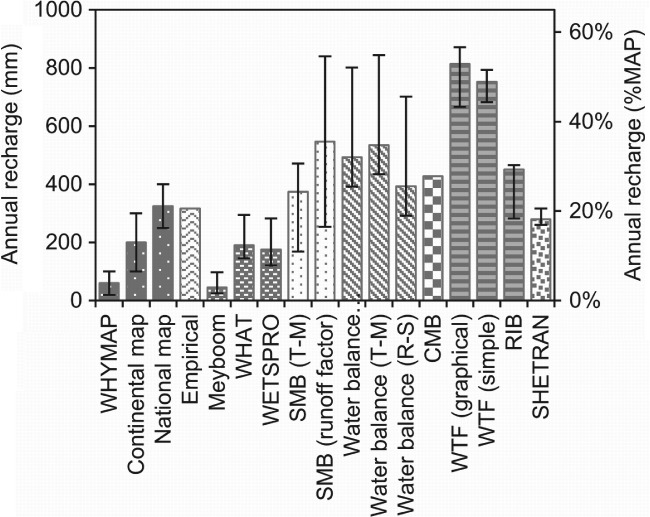
Median annual recharge estimates from all the techniques. The error bars give the interannular recharge range. *T‐M* = Thornthwaite‐Mather method of runoff or AET estimation. R‐S = Remote sensing method of AET estimation.

Recharge estimates from the various methods show high variability: 45‐814 mm/a or 3‐53%MAP (median annual precipitation) for the median annual recharge (Figure 5). The WHYMAP and Meyboom methods were rejected for this study with full reasoning provided in the Appendix S1.

### Sensitivity Analyses

**Figure 6:**
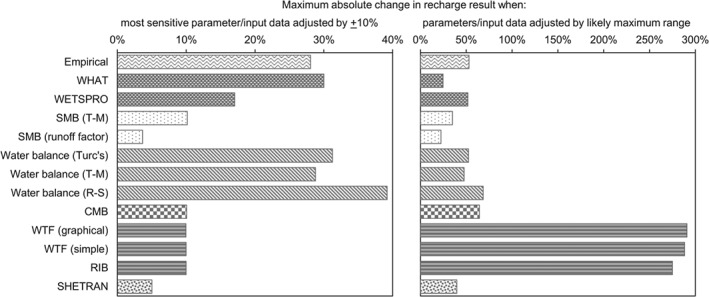
Comparison of the sensitivity of each recharge estimation method to ±10% adjustment in measured input data and modeling parameters (left) and range of uncertainty when the maximum likely deviations are applied (right).

Measured input data and modeling parameters were individually adjusted by ±10% to assess sensitivity. For some methods, only measured input data could be adjusted, for example, rainfall or groundwater level fluctuation. For other methods, it was possible to adjust modeling parameters determined during additional investigations or by “expert opinion,” for example, the recession constant for WHAT and WETSPRO analysis. In addition, to suggest the range of uncertainty, recharge was computed by each method using the likely maximum deviation in parameter values. Table 3 details the parameter adjustment and Figure 6 shows the sensitivity and uncertainty for each method.

**Table 3:** Parameters and Input Data Adjusted for the Sensitivity and Uncertainty Analysis

Method	Parameters/Input Data Individually Adjusted by ±10%. Most Sensitive Parameter in Italics	Maximum Likely Deviation of Parameters/Input Data Giving the Uncertainty Range
Empirical	*Annual average rainfall*	95% prediction interval from the rainfall‐recharge relationship curve
WHAT	River flow, BFI_max_, *recession constant*	Derived BFI_max_ and maximum/minimum recession constant that still gave an acceptable baseflow separation
WETSPRO	River flow, recession constant, *w*	Maximum/minimum recession constant and w that still gave an acceptable baseflow separation
SMB (T‐M)	Rainfall, PET, MC, LULC proportions, *% surplus to runoff*	Combined adjustment by ±10% of % surplus to runoff, MC and LULC proportions^1^
SMB (runoff factor)	Rainfall, PET, MC, LULC proportions, *runoff factor*	Combined adjustment by ±10% of runoff factor, MC and LULC proportions^1^
Water balance (Turc's)	*Rainfall*, temperature (for AET), runoff	Combined adjustment by ±10% of rainfall, temperature (for AET) and runoff^1^
Water balance (T‐M)	*Rainfall*, AET, runoff	Combined adjustment by ±10% of rainfall, AET and runoff^1^
Water balance (R‐S)	*Rainfall*, AET, runoff	Combined adjustment by ±10% of rainfall, AET and runoff^1^
CMB	Annual average rainfall, Cl_gw_, *Cl*_*wap*_	Measured range of Cl_wap_ (0.38‐1.12 mg/L)
WTF (graphical)	*Water level fluctuation, S*_*y*_	Measured range of S_y_ (0.05‐0.3)
WTF (simple)	*Water level fluctuation, S*_*y*_	Measured range of S_y_ (0.05‐0.3)
RIB	Water level fluctuation, rainfall, *S*_*y*_	Measured range of S_y_ (0.05‐0.3)
SHETRAN (phys. Based modeling)	Rainfall, PET, Strickler coefficient, S_y_, hydraulic conductivity, *layer thicknesses*, AE/PE ratio	Combined adjustment of layer thicknesses and AE/PE ratio by ±10%, and S_y_ and hydraulic conductivity within measured range that still gave an acceptable calibration

*BFI*_*max*_ = maximum value of long‐term ratio of baseflow to total streamflow; *w* = portion contributing directly to runoff; *PET* = Potential evapotranspiration; *MC* = root zone storage; *AE/PE* = actual to potential evaporation ratio. See methodological descriptions in the Appendix S1 for more information on these parameters.

1 The range in parameter/input data was uncertain, that is, there was no constraining measured range nor calibration targets.

[Corrections added on September 18, 2018, after first online publication: Last six sentences from Table 3 caption moved to main text].

The left plot in Figure 6 highlights the varying sensitivity of the methods. For example, it shows the water balance methods' high sensitivity to rainfall input and, essentially, lower sensitivity to any single parameter when the number of parameters increases (e.g., SMB and SHETRAN). The right plot in Figure 6 highlights the varying range of uncertainty in recharge result from different methods, which is dependent on the degree of uncertainty in the input parameters. For example, while the WTF and RIB methods show low sensitivity to a 10% variation in parameters, the recharge result has high uncertainty because the measured range in *S*_*y*_ was high; *S*_*y*_ is commonly uncertain due to the difficulties in accurate measurement and the heterogeneous nature of many aquifers. Uncertainty reduces with those methods that involve calibration, for example, WHAT and SHETRAN, as the maximum possible deviation in parameter values decreases. Additionally, when there is high uncertainty in input data, Figure 6 suggests which methods may be better selected.

## Discussion

### Reasons for the Range in Results

The range of recharge results presented in Figure 5 does not necessarily mean that some results are incorrect, as they need to be considered in the context of their spatiotemporal scale, what they represent and the limitations of each method. A recharge value that is comparatively high or low can provide insights on the conceptual model, especially if previously the conceptual model expected the method to provide an *actual* recharge estimate, and insights on uncertainty. A summary of the methods is provided in Table 4.

**Table 4:** Summary of Methods and Suggestions for Lessening Uncertainty in the Recharge Results

Method	Type of Recharge Computed	Under/Over Estimates^1^	Uncertainty^2^	How to Lessen Uncertainty^3^
WHYMAP	Actual	Under	Rejected because its scale is inappropriate for this study resulting in gross underestimation of recharge	
Continental map	Actual	Under	High	Use other methods
National map	Actual	Applicable	High	Use other methods
Empirical	Actual	Applicable	High	Increase number of recharge studies considered with greater geological, soils, vegetation and climate detail
Meyboom	Minimum	Under	Rejected due to problems of application on the study site hydrographs resulting in gross underestimation of recharge	
WHAT	Minimum	Under	Low	Utilize longer river flow time series and additional series from nested catchments
WETSPRO	Minimum	Under	Low‐medium	As above
SMB (T‐M)	Potential	Applicable	Medium	Increase rainfall and PET measurement density, utilize higher resolution soil and vegetation mapping and use a daily computation time step
SMB (runoff factor)	Potential	Over	Low‐medium	As above
Water balance (Turc's)	Actual	Over	Medium‐high	Increase rainfall and PET measurement density, utilize higher resolution vegetation mapping for better AET estimation, and use a daily computation time step
Water balance (T‐M)	Actual	Over	Medium	As above
Water balance (R‐S)	Actual	Applicable	Low‐medium	As above
CMB	Actual	Applicable	Medium	Increase rainfall chloride sampling frequency
WTF (graphical)	Change in storage	Over	High	Obtain more *S*_*y*_ estimates, utilize piezometers that are not biased toward good groundwater supply
WTF (simple)	Change in storage	Over	High	As above
RIB	Change in storage	Over	Medium‐high	As above
SHETRAN (phys. Based modeling)	Actual	Applicable	Low	Increase rainfall and PET measurement density, obtain more *S*_*y*_ and hydraulic conductivity estimates, and aquifer geometry measurements (e.g., with geophysics), utilize more river flow and groundwater level records for calibration

Note. It should be restated here that while the specific methods usually compute the specified type of recharge, this is particular to the conceptual model of the study site.

1 In comparison to the estimated actual recharge range for the study site of 280‐430 mm/a.

2 This relates to the sensitivity and uncertainty ranges in Figure 6 and the robustness of the method.

3 The suggestions present a best‐case scenario should time and budget allow.

As previously stated, unsaturated zone methods may overestimate recharge, explaining why the SMB methods applied here show high‐recharge values, that is, they are calculating potential recharge. The other uncertainty relates to which method to choose to determine the amount of runoff; application of a runoff factor based on measured river flows has lower uncertainty.

The streamflow hydrograph methods provide the lowest recharge estimates, supporting their classification as computing *minimum* recharge. While the Meyboom method was rejected (Appendix S1), the similarity of the WHAT and WETSPRO recharge results provides confidence in their *minimum* recharge estimates.

Considering the WTF and RIB methods, the suggestion by Healy and Cook (2002) that monitoring wells should be positioned in a “representative” location is reasonable for purposely installed piezometers, but hand‐dug wells will naturally be excavated where generations of experience indicate has good potential for groundwater abstraction, that is, there is a bias toward areas that receive lateral in addition to vertical recharge. It is unsurprising then that the WTF methods give the highest recharge estimates of all methods as they are actually computing the change in aquifer storage on a much smaller scale (10s of meters) than the other methods. The RIB method utilized the same groundwater level datasets and S_y_, though is constrained by the incorporation of a rainfall time series thus giving lower recharge estimates.

The empirical method is simple, but is built upon a substantial quantity of work by the authors of the studies used in the development of the method. However, confidence in the recharge result is low, due to several factors:

- Confidence in the quality of the published studies: The generation of the rainfall‐recharge relationship considered recharge estimates from all identified studies, even though 56% used only a single recharge estimation method and there was often uncertainty if the conceptual model meant applicability of assumptions or the type of recharge computed.
- Confidence in the transferability of the results: Figure S1 in Appendix S1 shows that the geographical distribution of the studies is biased to the Lake Tana Basin, Tigray, Dire Dawa, and around Addis Ababa. These four regions have specific rainfall intensity, evapotranspiration, hydrogeological and topographic characteristics that control the recharge rate.
- Confidence in the appropriateness of using annual rainfall total: Considering only the annual total rainfall fails to recognize the importance of rainfall intensity and distribution throughout the year. For example, a unimodal and a bimodal rainfall pattern would give different recharge rates even with the same annual total rainfall (Kingston and Taylor 2010).

The water balance methods should give *actual* recharge *if* the other fluxes are accurately quantified. While we may have a degree of confidence in values used for runoff and precipitation, AET is difficult to estimate, as the range in AET estimates from the three applied methods attests. The relatively high‐recharge estimates from the water balance methods are likely to be a symptom of underestimation of AET and greater uncertainty comes with decreasing robustness of AET estimation.

There is some uncertainty in the CMB recharge result due to the small number of rainfall chloride measurements and the assumption that chloride is not introduced into groundwater from any other source but precipitation. This assumption is valid at the study site regarding pollution and evaporites, which are not present, however, evapotranspiration from the saturated zone or from seepages that re‐infiltrate may cause an increase in the chloride concentration of groundwater. The discrepancy in recharge result of the CMB method may be because it averages over a longer period and larger area than the other applied methods.

SHETRAN modeling computes the change in aquifer storage for each cell, which becomes *actual* recharge when integrated over the catchment area as adjacent lateral inflows cancel. There is high confidence in these recharge estimates due to: substantial locally derived data was used to set up and calibrate the models as opposed to relying on just a few, potentially highly localized, input datasets or relying on averages; interannual variations in recharge totals correlate well between catchments with *r* = 0.81, and; recharge estimates are not sensitive to adjustments in individual parameter values. The spatially distributed nature of the model means that spatial variations in recharge due to lateral groundwater flow can be observed and understood, rather than providing misleading recharge estimations. Similarly, interannual variations in storage can be observed and measured rather than assumed to be negligible. However, this robustness of method depends on quantity and quality of data available for model setup, calibration and validation in addition to requiring a skilled operator with the necessary time available. Exploring the models' mass balances indicated why the SHETRAN recharge estimates are lower than those from other methods: recharge is reduced because, unlike other methods here presented, SHETRAN computes canopy and open water evaporation, both of which are significant at this site.

The map presented by Ayenew et al. (2008) was produced only at Ethiopian national‐scale and incorporates more local studies and experience than is possible with global or continental‐scale maps. Therefore, assuming that those local studies were conducted robustly, the national map gives a recharge estimate for which we have greater confidence.

It should be noted that only one of the nine alternative methods, the SHETRAN physically‐based modeling, involves calibration. This is the process of comparing predictions with the corresponding measured values and adjusting parameter values to achieve good agreement. SHETRAN was calibrated using river discharge and groundwater level time series data. The other recharge estimation methods do not have observed data against which to calibrate. For example, the water balance method utilizes observed rainfall, evapotranspiration and discharge data then solves for recharge. Calibration has been shown to reduce uncertainty, but this comes at the cost of complexity and increased data requirements. In general, the widely used methods of recharge estimation do not involve any calibration.

### Insights Gained on the Conceptual Model

The obvious insights gained from the multi‐method comparison were that not all methods were computing *actual* recharge or the results would be more similar (given similar spatiotemporal scale). Therefore, some assumptions must have been unsatisfied, which, rather than invalidating a method altogether, meant that the method was computing *potential* or *minimum* recharge or change in aquifer storage. Insights gained on the conceptual model mostly concern the amount and type of evapotranspiration, and the spatial variability of groundwater availability. High‐recharge values from the SMB methods indicate that all infiltration, which unsaturated zone methods are actually measuring, does not form recharge and there is likely to be interflow followed by discharge and/or evapotranspiration. The streamflow hydrograph methods' lowest recharge estimates indicate that groundwater is depleted prior to contributing to baseflow. Evapotranspirative losses from the saturated zone must be significant, which was thought likely given the shallow wet season water tables and spring/seepage‐fed inundated floodplains. High‐recharge values from the water balance methods are also suggestive that evapotranspiration may have been underestimated. Further evidence for this is the lower recharge estimate from SHETRAN that is due to its comprehensive simulation of canopy and open water evaporation and transpiration from the unsaturated and saturated zones resulting in greater total evapotranspiration losses. The high‐recharge values from the water table fluctuation methods, and high variability between wells, demonstrate the spatial variability in groundwater availability. The results show that groundwater flow, interflow and storage in certain areas can provide high potential for abstraction. Examples of other studies where fewer methods were applied and useful insights were gained are included in the Appendix S1.

### Recharge Estimate for Dangila *woreda*

Considering which types of recharge and spatiotemporal scales are relevant to this study, we restate the purpose as being to determine the resilience of shallow groundwater resources used for irrigation by rural communities in the Dangila area of Ethiopia; estimates of long‐term annual *actual* recharge at multiple catchment‐scales are therefore of primary interest. Although spatial assessments of aquifer storage change for small‐scale shallow aquifers, particularly at the seasonal‐scale, are also of significant interest to identify areas with the greatest potential for groundwater abstraction.

Considering the different types of recharge (Table 4), while the median recharge values from all of the methods used range from 45 to 814 mm/a, we expect that the long‐term *actual* recharge averaged over the general study area lies somewhere between the *minimum* and *potential* values of 176 and 547 mm/a, given by the lowest streamflow hydrograph and highest SMB methods, respectively. The range of median values given by all *actual* recharge methods is 279‐535 mm/a.

With regard to spatial scales, the methods based on groundwater level time series are highly localized and dependent on lateral inflows and other local factors, with values of recharge for individual wells from the RIB and the WTF methods ranging from under 100 to over 1600 mm/a. At the catchment‐scale, recharge values for the three catchments for each method used were generally consistent (Appendix S1), indicating some spatial consistency at this scale.

Having separated out and considered results by different types of recharge and spatial scales, determination of reliable *actual* recharge estimates for the general area around Dangila requires consideration of the confidence given to each relevant method. This can be based on factors discussed earlier, including: temporal representativeness of time‐series data; spatial representativeness of data; errors and uncertainties in input data; sensitivity of models to parameter values and input data; whether assumptions of methods are met. We have greatest confidence in the water balance method using the higher AET rate, the CMB method, and the SHETRAN modeling. Thus, we identify a reliable recharge range for the Dangila area of 280‐430 mm/a, which is consistent with the range from the national map (Ayenew et al. 2008).

## Conclusions

Nine methods, with a total of 17 variations, of groundwater recharge estimation were applied for a shallow aquifer in Ethiopia resulting in a wide range of median annual recharge values from 45 to 814 mm. This research shows that application of a range of methods may give a broad range of recharge values, but that it may not be necessary to discard results that appear to be outliers as these provide useful information. Consideration must be given to exactly what the “recharge” value represents: *potential*, *minimum*, or *actual* recharge, or change in aquifer storage. It is clear from the results presented that some methods providing estimates of *potential* recharge or storage change are likely to deliver overestimates of *actual* recharge while others that represent *minimum* recharge will deliver underestimates of *actual* recharge. Considering each method's spatiotemporal scale and uncertainty, we conclude that the most reliable recharge estimates for *actual* recharge in the general Dangila area are in the range 280 to 430 mm/a.

Insights gained from the multi‐method comparison study, including in particular assessment of results from methods where the usual assumptions were not strictly valid, enabled the hydrogeological system be better understood. First, by indicating that evapotranspiration is significant from (1) the saturated zone, and (2) the unsaturated zone following infiltration past the root zone due to interflow and seepage. Second, by revealing the spatial variation of the change in aquifer storage, which locally can be significantly higher than *actual* recharge estimates, giving further insight and confidence that areas could be identified with high potential for abstraction for small‐scale irrigation. Even though our recharge range is comparable to the national map results, we now have much higher confidence in the results and better understanding of our catchments and aquifers from our analyses.

This study has demonstrated for an extensive range of commonly used recharge methods applied at a single site that, in addition to quantifying uncertainty of recharge estimations, results from multi‐method comparisons should be clearly interpreted in relation to the types of recharge and spatiotemporal scale they represent, but can also provide additional benefits through improved hydrogeological understanding.

## Authors' Note

The author(s) does not have any conflicts of interest to report.

## Supporting information


**Appendix S1.** Supporting Information.
**Table S1.** Methods of recharge estimation grouped by hydrological zone. Methods applied in this study are marked with *.
**Table S2.** Comparison of recharge estimates from large‐scale mapping/modeling studies.
**Table S3.** Details of the recharge estimation studies used to develop a new empirical recharge method for Ethiopia based on the rainfall‐recharge relationship. Note that multiple recharge results from the same study relate to different recharge estimation methods applied and/or to different catchments or areas of the study site. *CMB* = chloride mass balance method, *SMB* = soil moisture balance method, *SNNPR* = Southern Nations, Nationalities and Peoples' Region, *WTF* = water table fluctuation method.
**Table S4a.** Calculation of actual evapotranspiration (*AET*), soil moisture deficit and soil moisture surplus (from which 50% forms recharge) using the Thornthwaite and Mather (1955, 1957) method. The year 2000 has been selected and grassland LULC category (*MC* = 200 mm) as an example. All values are in mm.
**Table S4b.** Calculation of actual evapotranspiration (*AET*) with the application of a runoff factor, soil moisture deficit and soil moisture surplus (which is equated to recharge) using the Thornthwaite and Mather (1955, 1957) method. The year 2000 has been selected and grassland LULC category (*MC* = 200 mm) as an example. All values are in mm.
**Table S5.** Representative *MC* values and proportional coverage of LULC classes for Dangila *woreda*.
**Table S6.** Comparison of rainfall chloride concentrations with other studies.
**Table S7.** Details and statistics of the calibration and validation periods for the SHETRAN catchment models.
**Figure S1.** Location map of the study area with other *recharge study sites* identified in the literature review shown on the right (image source: Google earth; Imagery ©2017 DigitalGlobe).
**Figure S2.** The components of a streamflow hydrograph. Total flow is the sum of the three components, or the entire area below the *Overland flow* curve. The plot is a snapshot of the WETSPRO analysis of Kilti river flow.
**Figure S3.** Snapshot of the Brante and Kilti hydrographs showing uncertainties encountered with the Meyboom method.
**Figure S4.** Location map of the AMGRAF and ILSSI monitoring wells (a *kebele* is similar to a parish).
**Figure S5.** Groundwater hydrograph through the wet season and determination of water table rise for the WTF method. *MW1* refers to the groundwater level in monitoring well 1 from where this snapshot is taken. (*mbgl* = metres below ground level).
**Figure S6.** Graphical output of the RIB model showing observed rainfall, observed groundwater level fluctuation (*WLF*), simulated groundwater level fluctuation (*dh [rib]*) and computed recharge. This plot shows the simulation of monitoring well MW3.
**Figure S7.** Graphical comparison of annual recharge estimates from the catchment‐scale techniques separated into catchments. *T‐M* = Thornthwaite‐Mather method of AET estimation.
