# Supplementary material for: Insights From a Multi‐Method Recharge Estimation Comparison Study
Source: Ground Water. 2018 Jul 19;57(2):245–58. doi: 10.1111/gwat.12801 (PMC6849517; doi:10.1111/gwat.12801)
Supplement: Supplementary file 1 — Appendix S1. Supporting Information. Table S1. Methods of recharge estimation grouped by hydrological zone. Methods applied in this study are marked with *. Table S2. Comparison of recharge estimates from large‐scale mapping/modeling studies. Table S3. Details of the recharge estimation studies used to develop a new empirical recharge method for Ethiopia based on the rainfall‐recharge relationship. Note that multiple recharge results from the same study relate to different recharge estimation methods applied and/or to different catchments or areas of the study site. CMB = chloride mass balance method, SMB = soil moisture balance method, SNNPR = Southern Nations, Nationalities and Peoples' Region, WTF = water table fluctuation method. Table S4a. Calculation of actual evapotranspiration (AET), soil moisture deficit and soil moisture surplus (from which 50% forms recharge) using the Thornthwaite and Mather (1955, 1957) method. The year 2000 has been selected and grassland LULC category (MC = 200 mm) as an example. All values are in mm. Table S4b. Calculation of actual evapotranspiration (AET) with the application of a runoff factor, soil moisture deficit and soil moisture surplus (which is equated to recharge) using the Thornthwaite and Mather (1955, 1957) method. The year 2000 has been selected and grassland LULC category (MC = 200 mm) as an example. All values are in mm. Table S5. Representative MC values and proportional coverage of LULC classes for Dangila woreda. Table S6. Comparison of rainfall chloride concentrations with other studies. Table S7. Details and statistics of the calibration and validation periods for the SHETRAN catchment models. Figure S1. Location map of the study area with other recharge study sites identified in the literature review shown on the right (image source: Google earth; Imagery ©2017 DigitalGlobe). Figure S2. The components of a streamflow hydrograph. Total flow is the sum of the three components, or the entire area below the Overlan [file GWAT-57-245-s001.pdf]

Insights from a multi-method recharge estimation comparison study

Supporting Information (SI) – Groundwater Journal

Authors and affiliations

David Walker[a]*, Geoff Parkin[a], Petra Schmitter[b], John Gowing[c], Seifu Admassu Tilahun[d], Alemseged Tamiru Haile[b], Abdu Yimer Yimam[d]

[a] School of Engineering, Newcastle University, UK

[b] International Water Management Institute (IWMI), Addis Ababa, Ethiopia

[c] School of Agriculture, Food and Rural Development, Newcastle University, UK

[d] Faculty of Civil and Water Resources Engineering, Bahir Dar Institute of Technology, Bahir Dar University, Ethiopia

* Corresponding author email: d.w.walker1@newcastle.ac.uk

Abstract

Although most recharge estimation studies apply multiple methods to identify the possible range in recharge values, many do not distinguish clearly enough between inherent uncertainty of the methods and other factors affecting the results. We investigated the additional value that can be gained from multi-method recharge studies through insights into hydrogeological understanding, in addition to characterising uncertainty. Nine separate groundwater recharge estimation methods, with a total of 17 variations, were applied at a shallow aquifer in northwest Ethiopia in the context of the potential for shallow groundwater resource development. These gave a wide range of recharge values from 45 to 814 mm/a. Critical assessment indicated that the results depended on what the recharge represents (actual, potential, minimum recharge or change in aquifer storage), and spatial and temporal scales, as well as uncertainties from application of each method. Important insights into the hydrogeological system were gained from this detailed analysis, which also confirmed that the range of values for actual recharge was reduced to around 280-430 mm/a. This study demonstrates that even when assumptions behind methods are violated, as they often are to some degree especially when data are limited, valuable insights into the hydrogeological system can be gained from application of multiple methods.

Contents

1. **Non-selection of certain recharge estimation methods**
2. **Recharge estimates for the study site from large-scale mapping and modelling**
3. **Field data not described in the main text that was used to develop the conceptual model and to parameterise models**
4. **Empirical method**
5. **Streamflow hydrograph methods**
6. **Soil moisture balance (SMB)**
7. **Basin water balance**
8. **Chloride mass balance (CMB)**
9. **Water table fluctuation (WTF) and rainfall infiltration breakthrough (RIB)**
10. **SHETRAN modelling**
11. **Comparison of recharge results from the three nested catchments**
12. **Insights gained on the conceptual model in other recharge studies**
13. **References (supporting information)**

1. Non-selection of certain recharge estimation methods

Recharge estimation methods can be subdivided to the three hydrological zones, namely surface water, the unsaturated zone and the saturated zone (Scanlon et al. 2002) with a further category for methods that consider all zones (Table S1).

Table S1. Methods of recharge estimation grouped by hydrological zone. Methods applied in this study are marked with *.

All zones	Surface water	Unsaturated zone	Saturated zone
*Basin water balance	Channel water budget	*Soil moisture balance (SMB)	*Water table fluctuation (WTF)
*Numerical modelling	*Streamflow hydrograph methods	Lysimeters	*Rainfall infiltration breakthrough model (RIB)
*Empirical methods, e.g. rainfall-recharge relationship	Seepage meters	Zero flux plane	Darcy's law
Remote sensing, e.g. GRACE, InSAR	Heat tracers	Infiltration models	Groundwater dating, e.g. tritium or CFCs
*Large-scale mapping	Isotopic tracers	Applied tracers, e.g. bromide or coloured dye	*Environmental tracers, e.g. chloride mass balance (CMB)
	[a] Numerical modelling	Historical tracers, e.g. tritium or ^{36}Cl	[a] Numerical modelling
		Environmental tracers, e.g. chloride	
		[a] Numerical modelling	
		Empirical methods, e.g. infiltration coefficients	

[a] SHETRAN modelling used in this study couples surface and subsurface (both unsaturated and saturated zones) flows.

All the techniques presented in Table S1 were explored and several were rejected for this study for the following reasons:

- Insufficient stream gauge locations were available for the channel water budget method.
- There are no surface water bodies available for seepage meter and thermocouple (for the heat tracer method) installation with the exception of highly seasonal rivers in which installed equipment would not be secure during wet season floods.
- Stable isotopes are very useful for providing information on recharge sources but not for quantitative recharge estimation (Healy, 2010).
- Lysimeters and zero flux plane equipment were excluded due to their high cost and maintenance requirements.
- Insufficient soil properties data were collected for use in infiltration models or to estimate infiltration coefficients.

- Applied tracers were not used due to the potential cultural conflicts that could arise from application of (albeit, harmless) chemicals into groundwater that is used domestically.
- The high rainfall and shallow thin aquifer leads to low groundwater residence times, therefore bomb-pulse tritium and ^{36}Cl are unlikely to still be present.
- There is insufficient spatial groundwater level and hydraulic conductivity data to determine hydraulic gradient for application of Darcy's law.
- Sampling groundwater for CFCs requires zero atmospheric contact (Gooddy *et al.*, 2012), which is impossible to achieve from open wells.
- Satellite based remote sensing is increasingly used to evaluate changes in groundwater storage, in particular with the Gravity Recovery and Climate Experiment (GRACE) satellite mission (Tapley *et al.*, 2004), and Interferometric Synthetic Aperture Radar (InSAR) (Galloway *et al.*, 1998). InSAR was not considered for this study, as its application is most suited to arid and semi-arid areas (Galloway and Hoffmann, 2007). GRACE measures temporal variations in the Earth's gravity field, which are used to estimate changes in terrestrial water storage. It has a spatial resolution of ~300 km and a monthly temporal resolution (NASA, 2016), both of which were considered too coarse for this study.

It can be seen in Table S3 that none of the methods rejected by this study was used in other Ethiopia recharge studies, with the exception of tritium analysis, though this was used for source assessments and timing rather than quantification (e.g. Demlie *et al.* (2007); Girmay *et al.* (2015)). However, there may be some bias as many of the studies share authors, or supervisors in the case of university theses.

2. Recharge estimates for the study site from large-scale mapping and modelling

There have been several attempts to produce global scale groundwater recharge maps beginning with L'vovich (1979), whose map was based on the estimation of the baseflow component of observed river discharge. More recently, Döll and Flörke (2005) introduced recharge into their WaterGAP Global Hydrological Model (WGHM). The authors state a shortfall of the model in that it is calibrated against measured river discharge only, due to the unavailability of direct recharge measurements. The scarcity of independent recharge estimates meant the model was tuned in arid and semi-arid areas against just twenty-five recharge assessments (nine of which were in sub-Saharan Africa) using chloride and isotope profiles. The WGHM was updated by Döll and Fiedler (2007) and incorporated into the World-wide Hydrogeological Mapping and Assessment Programme (WHYMAP, 2016) by BGR (the German Federal Institute for Geosciences and Natural Resources) and UNESCO. MacDonald *et al.* (2012) used WHYMAP to produce the recharge map in their "Quantitative maps of groundwater resources in Africa". The recharge estimates for the study site from WHYMAP and the other large-scale studies are

shown in Table S2. The Africa-wide recharge map presented by Altchenko and Villholth (2015) utilised data from the PCR-GLOBWB global hydrological model (van Beek *et al.*, 2011), which is calibrated against river discharge and reanalysis evapotranspiration data. At national scale, Ayenew *et al.* (2008) produced a hydrogeological framework of Ethiopia based on analysis of pertinent information from governmental and non-governmental organisations and academic institutions, as well as over a decade of field study comprising conventional hydrogeological mapping, well drilling, geophysics, hydrochemical and environmental isotope analysis and remote sensing techniques.

Table S2. Comparison of recharge estimates from large-scale mapping/modelling studies

Source	Scale	Resolution	Recharge
WHYMAP (2016)	Global	0.5^o	20-100 mm/a
Altchenko and Villholth (2015)	Africa	0.5^o	100-300 mm/a
Ayenew et al. (2008)	Ethiopia	~40 km*	250-400 mm/a

* Estimated from the map zonation.

The large-scale mapping and modelling methods share two characteristics that lessen confidence in those recharge results: 1) a necessarily low spatial resolution, and 2) the scarcity of recharge studies in the sub-Saharan Africa region that would aid calibration. Concerning the first point, at the resolution of 0.5^o for WHYMAP and the continental recharge map, it is unsurprising that the recharge estimates are dissimilar to those utilising catchment and local-scale methods. This region of Ethiopia has high variation in elevation, topography and climate, from 1100 m and hot dry plains immediately to west of Dangila, to cold wet mountains over 3000 m to the east (Fig. 1 of manuscript). These environments often appear in the same grid square at these scales. The particular usefulness of the maps in data scarce regions coincides with a higher uncertainty in those regions as products frequently cannot be validated. This point is valid not just for recharge but remote sensing and reanalysis weather data products (Walker et al. 2016). The global-scale WHYMAP clearly underestimates recharge and was rejected from this study.

3. Field data not described in the main text that was used to develop the conceptual model and to parameterise models

Geological surveys, water point surveys and workshops with the local community

The soils and aquifer properties for input into SHETRAN relied on field investigations as soil and geological mapping of the area is not available at sufficiently high resolution. The observed variation in well depths, differences in monitored well responses, variation in well pumping/recovery test

results, geological and soil observations, and discussions with local communities led to the definition of hydrogeological zones.

Outcrops are visible in riverbeds, occasionally on steeper slopes and in a few man-made excavations. The basalts are variously massive, fractured and vesicular with variations occurring in short distances. Above the solid geology lies weathered basalt regolith, itself overlain by red clayey loam soils. Local communities report that there are rarely problems with well sidewall collapse. Often the solid geology is reached abruptly and well excavation is halted. Depth to the top of the solid geology is variable. Wells are typically excavated until further excavation becomes impossible, therefore, the location of rockhead can be inferred from well depth. Over the three field visits, 80 wells were measured for estimation of regolith thickness; more wells were visited but access for measurement, such as in the case of wells fitted with handpumps, was not always possible. Rockhead was generally found to be deeper in more steeply sloping areas and shallower in floodplains. Rivers have often incised to the level of the rockhead where solid basalt forms the riverbed with banks of only 1 to 3 m in height.

In addition to season, topography appears to govern shallow groundwater availability. The variations in geology are sufficiently subtle, particularly concerning the regolith, which forms the shallow groundwater aquifer, to be less of a control on the hydrogeology than geomorphology. Near the end of the dry season in March/April within the floodplains where the solid geology is at a depth of around 4 m, the water table lies at 2-4 mbgl. The water table can often be seen as a seepage face at this depth within riverbank sections in floodplain sediments. However, on the larger and steeper slopes where rockhead is around 15 m deep the water table is at a depth of 12-15 m. Thus, the shallow aquifer is thicker on slopes giving deeper water tables and generally greater saturated aquifer thickness.

It is noted that farmers often talk of a well excavation striking rock at a shallow depth and being dry, then when the well is relocated a short distance away (~10 m) rock is struck at greater depth and the well fills with water. Such a situation is commonly ascribed to heterogeneous rockhead, however, the unsuccessful wells are perhaps more likely to be due to the presence of large and massive basalt boulders lying higher in the weathered profile as are often visible in riverbank sections.

Over 200 hand-dug wells were surveyed during field visits; the surveys included GPS location, depth and water level measurements, description of geology, topography, land use, pump/lifting device and cover, in-situ measurement of water temperature, pH and electrical conductivity, and discussions with local community over the well's use, seasonality and history. Water point surveys also included assessment of springs, many of which are used by the local community, whether developed or not, to collect water for domestic and potable use. Where springs and seepages emerge from gullies they

commonly occur at contacts between regolith and bedrock or gravelly regolith and more solid regolith. Springs and seepages are also very common around the edges of floodplains where the water table from the surrounding slopes intercepts the ground surface.

Hydrochemistry and stable isotope sampling, radon-222 measurements

The conceptual model was developed with the aid of hydrochemistry investigations. Samples of shallow and deep groundwater, surface water and rainwater were analysed in the laboratory for major ions, some trace elements, and stable isotopes oxygen-18 and deuterium ($\delta^{18}O$ and $\delta^{2}H$). In-situ testing involved measurement of pH, electrical conductivity (EC), total dissolved solids (TDS), temperature, and radon-222 concentration.

The shallow groundwater is consistent in chemistry both spatially and temporally. Residence time is low, indicated by low EC and ionic concentrations, suggesting that the resource could be vulnerable to drought. Surface water and shallow groundwater belong to the “bicarbonate calcium” type typical of recent recharge. The deep groundwater is of “bicarbonate sodium” type indicative of higher mineralisation due to longer residence time and greater distance of flow. The shallow groundwater samples from the wet season are very similar in chemistry to surface water samples indicating a high degree of and rapid interconnectivity. This was expected in the wet season from the observed very shallow water table. There is no hydrochemical evidence to suggest mixing between the shallow and deep groundwaters; they belong to clearly different water types. What’s more, Radon-222 measurements showed the opposite of what would be expected if surface water and shallow groundwater were being drawndown by abstraction from the deep boreholes: ^{222}Rn concentrations would be lower in the vicinity of the abstracting boreholes as groundwater discharge would be prevented but the reverse was measured. Radon-222 measurements did suggest that the large floodplains in river valleys are areas of groundwater discharge from the shallow regolith aquifer, whereas the narrower valleys with basalt riverbeds are not discharge areas.

Vegetation properties

Categorisation of vegetation was required for the SHETRAN models. The three categories were:

- Grassland – floodplains that are almost always and entirely utilised as pasture.
- Arable – where the majority of land is devoted to rainfed agriculture. The crops planted are 87% cereals (39% maize, 35% *tef* and 13% millet), with the rest being pulses, oilseeds, sugarcane, potatoes, vegetables, fruits, onions, garlic, and tomatoes (Belay and Bewket, 2013), the latter few generally occupying backyard plots.

- Shrub – areas of higher gradients with characteristic scrub-like vegetation.

How SHETRAN converts the potential evapotranspiration time series into actual evapotranspiration is specific for each of these categories. Each category within SHETRAN differs in rooting depth and root density at different depths, ground coverage at maximum seasonal extent, canopy storage capacity, leaf area index, and AET/PET at particular soil moisture tensions. These parameter values were estimated from ground observations in order to generalise vegetation and crop types followed by consultation of the key instructional texts for calculating water demand; FAO24 (Doorenbos and Pruitt, 1975) and FAO56 (Allen *et al.*, 1998), and other published studies providing detail of particular vegetation types, particularly, Canadell *et al.* (1996); Dardanelli *et al.* (1997); Cain (1998) and; Fan *et al.* (2016).

4. Empirical method

There are numerous published examples of the development of a rainfall-recharge relationship, often utilising secondary literature sources e.g. Bonsor and MacDonald (2010), Crosbie *et al.* (2010), Zhang *et al.* (1999). A series of Boolean searches were undertaken in February 2017 on Google Scholar and repeated on Web of Science in order to identify quantitative recharge studies from Ethiopia. The common Google search engine was also utilised to identify reports and other documents that may be outside of the usual scientific literature. The initial search terms were "groundwater AND recharge AND Ethiopia", which produced 1000s of documents. Despite including the search term "Ethiopia", many of the "hits" were from other countries and could be discarded immediately. Many other studies that did take place in Ethiopia had mention of recharge but had no recharge estimate and were also discarded. The first 150 hits were reviewed for inclusion in the analysis before the searches became more specific by adding a recharge estimation method in an attempt to exclude the non-quantitative recharge studies. Specific searches involved adding a fourth term to the search described above. The added search terms were (independently): "baseflow", "chloride", "balance", "fluctuation", "wtf", "tritium", "lysimeter", "zero flux plane", "channel water budget", "seepage meter", "heat tracer", "Darcy's law", "infiltration model", "bromide", "applied tracer", "isotropic tracer", "infiltration coefficient" and "CFC". The first 100 documents were reviewed for each search, or all the documents if there were fewer than 100 hits. In an attempt to fill in the gaps when the study site locations were plotted on a map of Ethiopia (Fig. S1), further searches were conducted with a geographic location replacing the third search term (the first two still being "groundwater AND recharge"). The geographic search terms used were the regions of Ethiopia that were under or not represented on the map of study sites, namely: "Gambella", "Benishangul Gumuz", "Afar", "Somali AND Ethiopia" and "SNNPR". The first 50 documents were reviewed, seeing as, by this stage of the search, the majority of hits had

been reviewed previously. Table S3 presents information on all of the studies identified in addition to the reference. Several studies are included that were found during other literature searches undertaken for other aspects of the wider AMGRAF (Adaptive Management of shallow GRoundwater for small-scale irrigation and poverty alleviation in sub-Saharan AFrica) project. Where a study was cited within another study, an attempt was made to locate the cited study for review. Occasionally, a cited study could not be found though sufficient detail was provided for its inclusion in this project (listed in Table S3 as "… cited in…"). Several times a cited study with or without sufficient detail was not listed in the citing study's reference list and as such, after a failed attempt at location, it was not included in this project. Attempts were made within Ethiopia to access often-cited but seldom seen reports via university and other organisations' libraries with limited success. Forty-nine quantitative studies were located comprising 22 peer-reviewed articles, the rest being grey literature (predominantly MSc theses). Where a study used multiple methods or multiple catchments, recharge results were considered independently. Therefore, 102 annual recharge estimates could be plotted against annual rainfall. Various trendlines were fitted through the data, excluding points with rainfall below certain thresholds (in an Africa-wide study, Bonsor and MacDonald (2010) recognised a linear relationship between recharge and rainfall above 500 mm/a). A quadratic trendline, reflecting an increase in recharge disproportionate to increasing precipitation, achieved the best R^2 and standard error.

Table S3. Details of the recharge estimation studies used to develop a new empirical recharge method for Ethiopia based on the rainfall-recharge relationship. Note that multiple recharge results from the same study relate to different recharge estimation methods applied and/or to different catchments or areas of the study site. *CMB* = chloride mass balance method, *SMB* = soil moisture balance method, *SNNPR* = Southern Nations, Nationalities and Peoples' Region, *WTF* = water table fluctuation method.

**Location**	**Area (km^2)**	**Recharge estimation method**	**Annual rainfall (mm)** [a]	**Annual recharge (mm)** [a]	**Publication**	**Reference**
Raya Valley Basin	2579	Water balance<br>CMB	813	50<br>52	Addis Ababa University master's thesis	Abdella (2011)
Kobo Valley Basin, Tigray	1351	Water balance<br>CMB	813	63<br>53		
Gilgel Abay Catchment	4178	SWAT model	2141	753	Landscape Dynamics, Soils and Hydrological	Abiy *et al.* (2016)
Gumera Catchment	1418		1424	438		

Ribb Catchment	2132		1407	453	Processes in Varied Climates	
Megech Catchment, Lake Tana Basin, NW Ethiopia	661		1251	407		
Lower Awash Sub-Basin	41887	CMB	553	29	Addis Ababa University master's thesis	Addisu (2012)
Aysha Basin, Afar	4092		553	58		
Geba Basin, Tigray	5150	WetSpass model	550	22	KU Leuven (Belgium), master's thesis	Alene (2006) cited in Tesfagiorgis *et al.* (2011)
Negelle Borena, south Ethiopia	?	?	647	31	GSA (Geological Society of America) Annual Meeting presentation	Ali Jr (2006)
Teji Catchment, central Ethiopia	700	Streamflow hydrograph	1104	109	Addis Ababa University master's thesis	Andualem (2008)
		Water balance incorporating SMB and streamflow hydrograph		325		
Gilgel Abay, Koga and Kilti Catchments	1664	Streamflow hydrograph	1376	70	ITC (Netherlands), master's thesis	Asmerom (2008)
Megech Catchment	514	BASF rainfall-runoff model	959-1212	50-77		
Lake Tana Basin, NW Ethiopia	6316	CMB	1094-1730	45-155		

Weybo Catchment, SNNPR	574	Water balance incorporating SMB<br>Streamflow hydrograph (baseflow separation)<br>Streamflow hydrograph (Meyboom method)<br>CMB	1341	88<br>63<br>75<br>124	Addis Ababa University master's thesis	Aychluhim (2006)
Lake Tana Basin, NW Ethiopia	15339	"CMB, baseflow separation, etc" [b]	1094-1730	70-120	Addis Ababa University master's thesis	Ayalew (2010)
Meki Basin, central Ethiopia	3051	Streamflow hydrograph<br>WATBAL water balance model	762-1138	80<br>63	SINET: Ethiopian Journal of Science	Ayenew (2008)
Lake Awassa Basin, central Ethiopia	1455	MODFLOW model	1030	47	Lakes and Reservoirs: Research and Management	Ayenew and Tilahun (2008)
Raya Valley Basin, Tigray	2480	WATBAL water balance model	813	86	Water International	Ayenew *et al.* (2013)
Guder Sub-Basin	7088	SMB<br>CMB	1424	258<br>210	Journal of African Earth Sciences	Azagegn *et al.* (2015)
Muger Sub-Basin	8263	SMB<br>CMB	1201	150<br>163		
Jema Sub-Basin	6760	SMB<br>CMB	992	164<br>120		
Upper Awash Sub-Basin,	16000	SMB<br>CMB	1112	232<br>133		

central Ethiopia						
Southern Lake Tana Basin, NW Ethiopia	1664 [c]	Streamflow hydrograph	1541 [c]	308	Technical report for the Ministry of Water Resources (MoWR)	BCEOM (1998) cited in Ayalew (2010)
Kulubi area, Dire Dawa	?	Water balance using assumed runoff coefficients	626 [c]	150	Technical report for the Ministry of Water Resources (MoWR)	BCEOM (2005) cited in Tilahun and Merkel (2009)
Dengego area, Dire Dawa	?		626 [c]	50		
Between Melko Jebdu and Hurso, Dire Dawa, NE Ethiopia	260	Spring-area relationship	721	43		
Lake Beseka Basin, central Ethiopia	505	WTF	534	42	University of Bonn (Germany) PhD thesis	Belay (2009)
		EARTH modelling		47		
		CMB		1.2		
Upper Awash Sub-Basin	6735	Water balance	1077	131	Journal of Geoscience and Environmental Protection	Berehanu *et al.* (2017)
		CMB		135		
		Streamflow hydrograph		91		
		Infiltration model		157		
Muger Sub-Basin	1770	Water balance	1077	125		
		CMB		148		
		Streamflow hydrograph		158		
		Infiltration model		239		
Jema Sub-Basin, central Ethiopia	304	Water balance	1077	130		
		CMB		122		
		Streamflow hydrograph		86		
		Infiltration model		239		

Akaki Catchment, central Ethiopia	1500	CMB	1254	265 [d]	Hydrological processes	Demlie *et al.* (2007)
Akaki Catchment, central Ethiopia	1464	SMB<br>CMB	1254	105<br>273	Environmental Earth Sciences	Demlie (2015)
Fogera Plain, Lake Tana Basin, NW Ethiopia	500	Soil moisture profiles and groundwater level/evaporation relationship	1360	850-1000 [e]	Land Degradation & Development	Enku *et al.* (2016)
Adama-Wonji Basin, central Ethiopia	1760	MODFLOW model	860	123	Environmental Earth Sciences	Furi *et al.* (2011)
Werii, Tekeze Basin, Tigray	1797	WetSpa<br>WetSpass	717	30 [b]	Haramaya University (Ethiopia) master's thesis	Gebremeskel (2015)
Geba Basin, Tigray	5260	WetSpass model	400-950	41	Journal of Hydrology	Gebreyohannes *et al.* (2013)
Dire Jara and Hurso, Dire Dawa, NE Ethiopia	85-90	?	626 [c]	31	Technical report for the Ministry of Water Resources (MoWR)	Gibb and Seureca (1996) cited in Tilahun and Merkel (2009)
Dire Dawa, NE Ethiopia	?	?	626 [c]	40	Hebrew University, Jerusalem, PhD thesis	Greitzer (1970) cited in Tilahun and Merkel (2009)
Upper Wabe Sub-Basin, central Ethiopia	4489	SMB<br>Water balance<br>Streamflow hydrograph	924	21<br>23<br>155	Addis Ababa University master's thesis	Habtamu (2009)

Raya Valley Basin, Tigray	1085	CMB MODFLOW model	724	116 114	ITC (Netherlands), master's thesis	Hagos (2010)
Berga Catchment, central Ethiopia	303	Water balance incorporating SMB and streamflow hydrograph	1119	83	Addis Ababa University master's thesis	Hussen (2006)
Aynalem Wellfield, Mekelle, Tigray	104	CMB MODFLOW model	670	30-40 42	ITC (Netherlands), master's thesis	Kahsay (2008)
Gedeb Catchment, central Ethiopia	290	SWAT model	1392	467	Proceedings of 2012 international congress on environmental modeling and software managing resources of a limited planet, sixth biennial meeting, Leipzig, Germany	Koch *et al.* (2012)
Gidabo Basin, south Ethiopia	3302	SWAT model	800 1600	25 410	Journal of Hydrology: Regional Studies	Mechal *et al.* (2015)
Koraro Area, Tigray	59	Water balance incorporating SMB	549	57	Momona Ethiopian Journal of Science	Nedaw (2010)
Meki Basin, central Ethiopia	1669	Water balance incorporating SMB	992	117	Addis Ababa University master's thesis	Netsanet (2007)

Becho	1552	Water balance incorporating SMB<br>Streamflow hydrograph	1131	320<br><br><br>81	Addis Ababa University master's thesis	Nuramo (2016)
Koka, Upper Awash, central Ethiopia	1461	Water balance incorporating SMB<br>Streamflow hydrograph	879	50<br><br><br>104		
Koka<br>Becho, Upper Awash, central Ethiopia	1461<br>1552	Water balance incorporating SMB and streamflow hydrograph	900<br>1026	27<br>227	Addis Ababa University master's thesis	Reys (2016)
Shaya Watershed, SE Ethiopia	504	SWAT model	1071	174	HESS	Shawul *et al.* (2013)
Bulbul Basin, SW Ethiopia	508	Water balance incorporating SMB and streamflow hydrograph<br>Streamflow hydrograph	1520	350<br><br><br><br><br>395	Asian Journal of Applied Science and Engineering	Shimelis *et al.* (2014)
Upper Bilate Catchment, SNNPR	2075	Streamflow hydrograph<br>Water balance incorporating SMB and streamflow hydrograph	1232	129<br><br>96	Addis Ababa University master's thesis	Sintayehu (2009)
May Nugus Catchment, Tigray	15	Water balance incorporating SMB	738	19	International Journal of Earth Sciences and Engineering	Tadesse *et al.* (2010)

Illala Catchment, Tigray	340	WetSpass model	550	66	Momona Ethiopian Journal of Science	Teklebirhan *et al.* (2012)
Bilate Catchment, SNNPR	5625	Water balance incorporating SMB and streamflow hydrograph<br>Streamflow hydrograph	1146	116<br><br><br><br><br>201	Addis Ababa University master's thesis	Tesfaye (2010)
Dire Dawa, NE Ethiopia	920	WetSpass model	626	28	Hydrogeology Journal	Tilahun and Merkel (2009)
Zenako-Argaka Catchment, Tigray	4	MODFLOW model	724	167	Hydrogeology Journal	Vandecasteele *et al.* (2011)
Zenako-Argaka Catchment, Tigray	2	Runoff model<br>SMB model<br>MODFLOW model	"525-900 (average = 687)"	110-334 [b]	Hydrological Sciences	Walraevens *et al.* (2009)
Mendae Plain, Tigray	5	CMB<br>SMB model	512	18 [b]	Land Degradation and Development	Walraevens *et al.* (2015)
Aba'ala *woreda*, Afar	254	Hydrochemistry	340-480	43	Research and development experience on dryland husbandry in Ethiopia	Woldearegay (2004)
Aynalem and Illala Catchments, Tigray	?	?	576	53	Addis Ababa University master's thesis	Yihdego (2003) cited in Teklebirhan *et al.* (2012)
Aynalem Catchment, Tigray	?	MODFLOW model	550 [c]	61	Mekelle University master's thesis	Zeru (2008) cited in

						Teklebirhan *et al.* (2012)
Gilgel Abay Catchment	1640	Streamflow hydrograph	1614	379	Addis Ababa University master's thesis	Zewdie (2010)
Gumera Catchment	1394		1292	158		
Megech Catchment	492		1081	62		
Ribb Catchment	1592		1263	77		
Koga Catchment	302		1410	234		
Kilti Catchment, Lake Tana Basin, NW Ethiopia	698		1322	57		

[a] Where a range is given, the mean rainfall or recharge was used for the plot.

[b] It is uncertain which technique gave which result.

[c] Unclear from the study; therefore, the value is taken from a study in the same area.

[d] For uncertain reasons, this value is stated to be an overestimate.

[e] Not included in the analysis as the study-specific conceptual model renders the recharge value inapplicable.

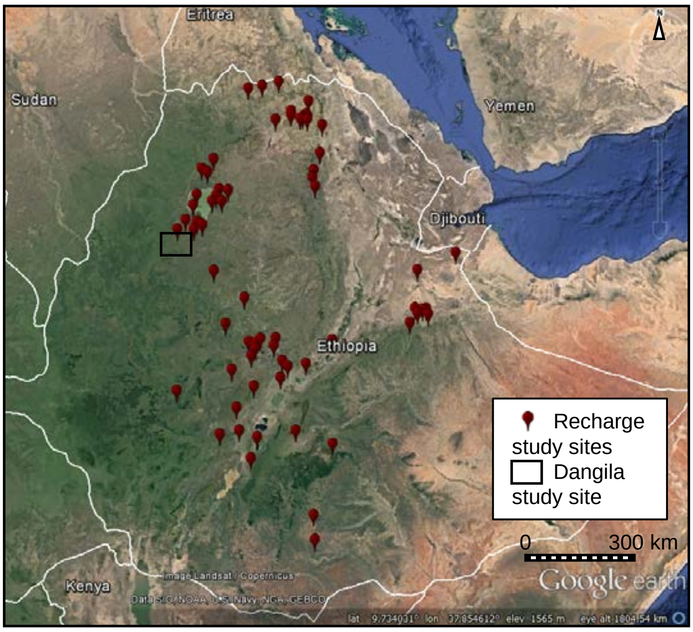

Fig. S1. Location map of the study area with other *recharge study sites* identified in the literature review shown on the right (image source: Google earth; Imagery ©2017 DigitalGlobe).

5. Streamflow hydrograph methods

Recharge estimation using streamflow hydrograph methods typically involves separating the baseflow component (Fig. S2) and approximating this to groundwater recharge. In humid regions, according to Döll and Fiedler (2007), who required recharge assessments for the entire globe for calibration of their WaterGap Global Hydrology Model, streamflow hydrograph methods are the most commonly applied recharge estimation method and numerous examples are available in the literature. The methods are idealised in assuming that groundwater storage remains constant interannually, or is in balance over longer time periods, and that precipitation entering the aquifer as recharge must be balanced by groundwater discharge into rivers that forms baseflow.

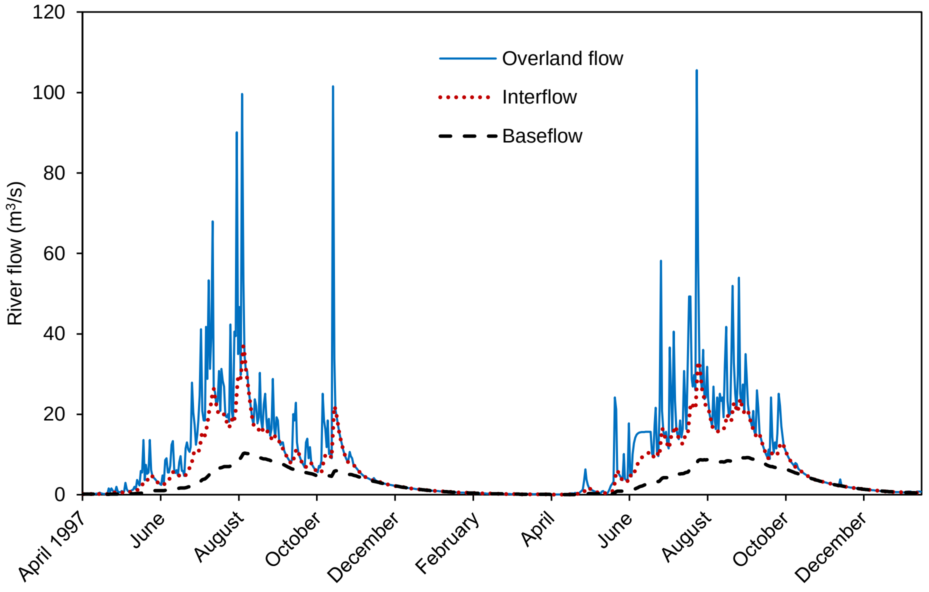

Fig. S2. The components of a streamflow hydrograph. Total flow is the sum of the three components, or the entire area below the *Overland flow* curve. The plot is a snapshot of the WETSPRO analysis of Kilti river flow.

Three streamflow hydrograph methods were used in this study, the baseflow recession method presented by Meyboom (1961), and two digital recursive filter tools, the Web GIS based Hydrograph Analysis Tool (WHAT) (Lim *et al.*, 2005) and WETSPRO (Willems, 2009). For both Meyboom and WHAT, the interflow is part of the quick flow component whereas for WETSPRO the interflow is separated in a two-step digital filter method after baseflow filtering. There are many very similar digital filter programs available for baseflow separation and two were chosen to assess whether they would give significant differences in recharge result. More in-depth comparison studies of baseflow separation methods are available, e.g. Chapman (1999), Eckhardt (2008).

The Meyboom method uses analysis of baseflow recession from at least two consecutive years to estimate recharge. The stream hydrograph is plotted on semi-logarithmic paper creating a straight-line recession curve. The start and end times of the recession "curve" are noted manually. According to Meyboom (1961), the total potential groundwater discharge can be estimated from

$$V_{tp} = \frac{Q_0 t_1}{2.3} \quad \text{(E1)}$$

where V_{tp} is the total potential groundwater discharge, Q_0 is the baseflow at the start of the recession, and t_1 is the time that it takes the baseflow to drop from Q_0 to 0.1 Q_0. The amount of potential baseflow, V_t, remaining at some time, t, after the initiation of baseflow may be estimated by:

$$V_t = \frac{V_{tp}}{10^{(t/t_1)}} \quad \text{(E2)}$$

The difference between the remaining potential groundwater discharge at the end of a given baseflow recession and the total potential groundwater discharge at the beginning of the next recession represents the recharge that takes place between these two recessions, i.e.

$$V_{tp} - V_t = R \quad \text{(E3)}$$

where R is the total quantity of recharge which is divided by the basin area to give a value in mm/a.

The Meyboom method is well-used, e.g. Mau and Winter (1997), Kumai and Mitamura (2004), Berhail *et al.* (2015), though seems inappropriate for this study site. The recharge quantities calculated using the Meyboom method are the lowest of all methods. Uncertainty of manually choosing start times of baseflow recessions could lead to recharge underestimation when early times of interflow recession have been incorrectly identified as baseflow recession (Fig. S3). Furthermore, when plotted on semi-log graph paper, baseflow recessions of the three rivers did not always form a straight-line meaning the Meyboom method is rejected for this study site.

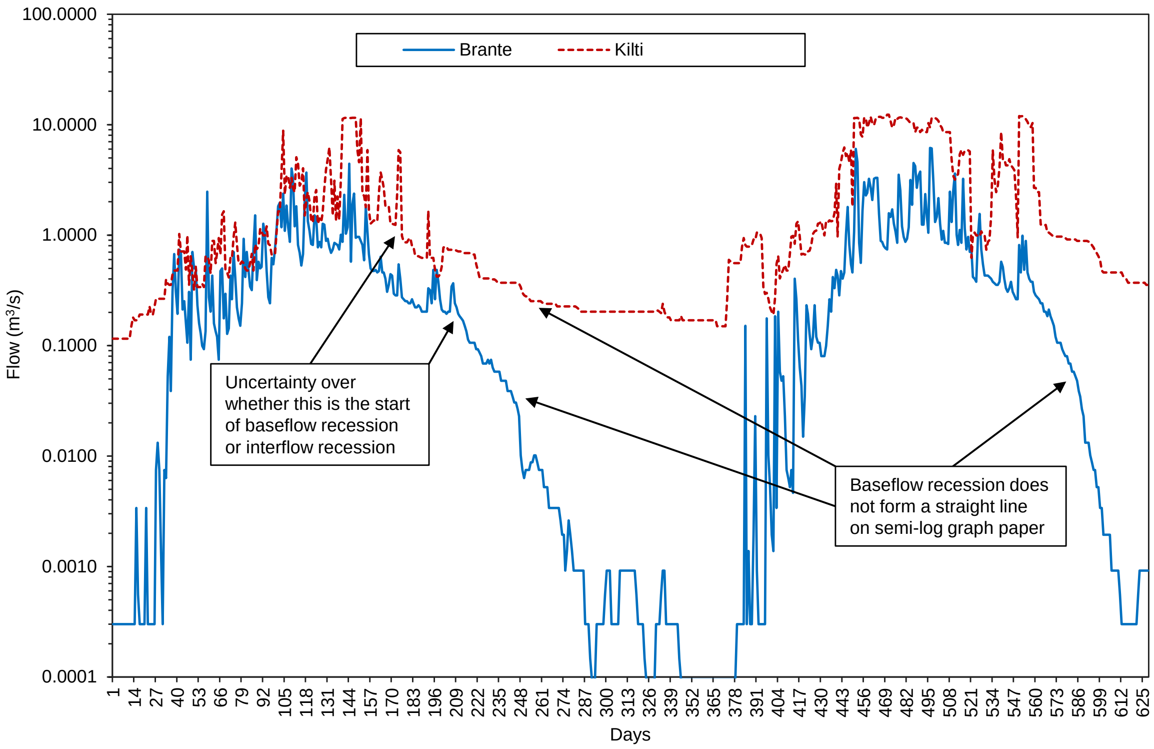

Fig. S3. Snapshot of the Brante and Kilti hydrographs showing uncertainties encountered with the Meyboom method

The WHAT method requires a filter parameter *a* (the recession constant) and a BFI_{max}, which is the maximum value of long-term ratio of baseflow to total streamflow. The developers of WHAT recommend using representative BFI_{max} values proposed by Eckhardt (2005): 0.80 for perennial streams with porous aquifers, 0.50 for ephemeral streams with porous aquifers, and 0.25 for perennial streams with hard rock aquifers. The local hydrological system meant a BFI_{max} of 0.50 was most appropriate for this study, which was confirmed by a calculated BFI_{max} 0.57 (average of the three catchments). The WETSPRO method uses two main parameters to filter out the baseflow and interflow respectively. For each of the two components a recession constant *k*, which relates to the WHAT filter parameter as $a = \exp(-1/k)$, and the portion contributing directly to runoff *w* are defined. Both parameters are dependent on catchment size and characteristics.

6. Soil moisture balance (SMB)

The SMB method has the advantage over other unsaturated zone methods that it is not point based, rather it is at the scale that the precipitation, potential evapotranspiration (PET) and soil property inputs remain applicable. Rainfall totals were utilised in addition to the required meteorological time series to enable PET computation using the Penman-Monteith FAO-56 method (Allen *et al.*, 1998). The Thornthwaite-Mather (1955, 1957) (T-M) method additionally requires a value for soil moisture

retention (*MC*), which is dependent upon vegetation and soil type. *MC*, or 'root zone storage', equals field capacity multiplied by depth of root zone. *MC* values were assigned to each LULC class and recharge was calculated individually for each class (Tables S4 and S5). The SMB method calculates total monthly actual evapotranspiration (AET) and soil moisture surplus with this surplus contributing to recharge. The direct runoff component is dealt with in two ways:

(a) It can be subtracted from the precipitation input by applying a runoff factor that could be taken from literature (e.g. Bakundukize *et al.* (2011)), derived from streamflow hydrograph analysis (e.g. Demlie (2015)), or modelled (e.g. Walraevens *et al.* (2009)).

(b) A portion of the soil moisture surplus is subtracted; Thornthwaite and Mather (1957) recommend subtracting 50 % (e.g. Chishugi and Alemaw (2009), Azagegn *et al.* (2015).

Both methods were applied in this study; the runoff factor based on a simple flow separation (IOH, 1980) conducted on the longer time series river flow records (Kilti and Amen).

Calculation of groundwater recharge using a SMB involved the application of the Thornthwaite and Mather (1955, 1957) method and equating soil moisture surplus to recharge. The SMB calculations are shown in Table S4 with a description of the parameters below.

Table S4a. Calculation of actual evapotranspiration (*AET*), soil moisture deficit and soil moisture surplus (from which 50 % forms recharge) using the Thornthwaite-Mather (1955, 1957) method. The year 2000 has been selected and grassland LULC category (*MC* = 200 mm) as an example. All values are in mm.

	J	F	M	A	M	J	J	A	S	O	N	D	Annual total
**P**	0	7.1	7.5	77.0	135.3	344.1	313.0	436.1	237.8	265.3	70.0	2.5	1895.7
**PET**	98.7	105.1	117.4	119.1	114.5	86.3	81.3	84.6	90.1	93.4	92.4	90.0	1172.7
**P-PET**	-98.7	-98.0	-109.9	-42.1	20.8	257.8	231.7	351.5	147.7	172.0	-22.4	-87.5	
**APWL**	-208.6	-306.6	-416.5	-458.5	0	0	0	0	0	0	-22.4	-109.9	
**SM**	70.5	43.2	24.9	20.2	41.0	200.0	200.0	200.0	200.0	200.0	178.8	115.5	
**ΔSM**	-45.0	-27.3	-18.3	-4.7	20.8	159.0	0	0	0	0	-21.2	-63.4	
**AET**	45.0	34.4	25.8	81.7	114.5	86.3	81.3	84.6	90.1	93.4	91.2	65.9	894.0
**SMD**	53.7	70.7	91.6	37.3	0	0	0	0	0	0	1.2	24.1	
**SUR**	0	0	0	0	0	98.8	231.7	351.5	147.7	172.0	0	0	1001.7
**Rech.**	0	0	0	0	0	49.4	115.9	175.8	73.9	86.0	0	0	500.9

Table S4b. Calculation of actual evapotranspiration (*AET*) with the application of a runoff factor, soil moisture deficit and soil moisture surplus (which is equated to recharge) using the Thornthwaite-Mather (1955, 1957) method. The year 2000 has been selected and grassland LULC category (*MC* = 200 mm) as an example. All values are in mm.

	J	F	M	A	M	J	J	A	S	O	N	D	Annual total
P_{eff}	0	0.2	10.5	26.9	131.3	230.8	290.4	316.5	208.4	83.4	17.1	1.3	1316.7
**PET**	98.7	105.1	117.4	119.1	114.5	86.3	81.3	84.6	90.1	93.4	92.4	90.0	1172.7
**P-PET**	-98.7	-104.9	-106.9	-92.2	16.7	144.5	209.1	231.9	118.3	-9.9	-75.3	-88.7	
**APWL**	-262.7	-367.6	-474.5	-566.7	0	0	0	0	0	0	-75.3	-164.0	
**SM**	53.8	31.8	18.6	11.8	28.5	173.0	200.0	200.0	200.0	200.0	137.3	88.1	
**ΔSM**	-34.3	-21.9	-13.2	-6.9	16.7	144.5	27.0	0	0	0	-62.7	-49.2	
**AET**	34.3	22.1	23.7	33.8	114.5	86.3	81.3	84.6	90.1	93.4	79.8	50.5	794.3
**SMD**	64.4	83.0	93.7	85.3	0	0	0	0	0	0	12.5	39.5	
**SUR**	0	0	0	0	0	0	182.1	231.9	118.3	0	0	0	532.3
**Rech.**	0	0	0	0	0	0	182.1	231.9	118.3	0	0	0	532.3

Where

P = monthly rainfall total as measured by the NMA (National Meteorological Agency of Ethiopia) at the meteorological station in Dangila town, in the above examples for the year 2000.

P_{eff} = monthly effective rainfall, which is the monthly rainfall total minus the direct runoff computed by a simple flow separation (IOH, 1980) applied to river flow data; $P_{eff} = P.(1-RF)$ where *RF* is the runoff factor calculated to be 0.145.

PET = potential evapotranspiration calculated using the Penman-Monteith FAO-56 method (Allen *et al.*, 1998) with input parameters measured by the NMA at Dangila, in the above examples for the year 2000.

APWL = accumulated potential water loss; the summation begins with November, the first month of the dry season, until end April.

MC = moisture capacity; also known as soil moisture retention and equals field capacity multiplied by depth of root zone. It is dependent upon vegetation and soil type and, in the above examples, is specified as 200 mm for the grassland land use/land cover (LULC) category (see further explanation below).

SM = soil moisture; the soil moisture during dry months is obtained using accumulated potential water loss by the following formula: $SM = MC.\exp(APWL/MC)$. For the wet months, the soil moisture is

calculated by adding the excess rainfall of the current month to the soil moisture of the previous month; where this exceeds the moisture capacity, the excess is booked as moisture surplus.

ΔSM = change in soil moisture from the previous month.

AET = actual evapotranspiration; for the wet months *AET* = *PET* as it is assumed that all rainfall is available for plants. During dry months, *AET* = *P* + moisture loss from the soil (i.e. negative *ΔSM*).

SMD = soil moisture deficit; the difference between *PET* and *AET*.

SUR = surplus moisture available for infiltration. According to Thornthwaite and Mather (1955, 1957) and shown in Table S4a, 50% of *SUR* = recharge (*Rech*). Or, where a runoff factor is applied, shown in Table S4b, 100 % of surplus moisture forms recharge (*SUR* = *Rech*).

Rech. = recharge

MC values were assigned to each LULC class and the recharge was calculated for each. The recharge values were then multiplied by the proportional area of each LULC class and summed to give the recharge for Dangila district. The LULC information was taken from ADSWE (2015) and the *MC* values were assigned based on field investigations of soil and vegetation types (Table S5).

Table S5. Representative *MC* values and proportional coverage of LULC classes for Dangila *woreda*.

LULC class	Coverage (%)	MC (mm)
Built up	8.72	10 [a]
Cultivated	71.7	150
Forest	11.0	300
Grassland	7.9	200
Shrub and bush	0.5	250

[a] The built up areas have some patches of vegetation; therefore, a nominal *MC* was applied.

7. Basin water balance

The water balance, or water budget, was the most commonly used method identified during the literature review of Ethiopian recharge studies (see Table S3 for examples). AET is not straightforward to estimate and was calculated with three methods for comparison: (1) The T-M method mentioned in the previous section (Table S4); (2) Application of Turc's formula (Turc 1954), see below for a full description, and; (3) A value estimated by Allam *et al.* (2016) for this region of the Tana Basin by combining remote sensing and river flow records. The average AET values were 789, 831 and 931 mm/a, respectively. Annual average runoff values were obtained using a simple and standard flow separation method (IOH 1980); the separated baseflow was subtracted from total flow to give the

direct runoff component. The basin water balance can be increased in complexity by computing at a daily time step and utilising high-resolution soil and vegetation mapping with which to calculate AET; such data were not available for this study. Accurate quantification of all the fluxes is always troublesome though is required in order to leave an accurate residual that is equated to *actual* recharge (Scanlon et al. 2002). There persists the potential for unaccounted groundwater depletion as described in the streamflow hydrograph section.

Turc's formula requires only annual precipitation and temperature and was established empirically based on 254 watersheds, globally distributed (including 10 in Ethiopia) in different climatic zones (Turc, 1954). Actual evaporation from a catchment, *AET*, is defined as

$$AET = P/((0.9 + (P^2/L^2))^{-0.5}) \quad \text{(E4)}$$

Where *P* is average annual precipitation in mm and

$$L = 300 + 25T + 0.05T^3 \quad \text{(E5)}$$

Where *T* is average annual air temperature in ^{o}C.

8. Chloride mass balance (CMB)

The CMB method requires: P_{eff} – the average annual effective precipitation (rainfall minus direct runoff), Cl_{wap} – the weight-average chloride concentration in precipitation including dry deposition, and Cl_{gw} – the average chloride concentration in groundwater. Direct runoff was calculated using a simple flow separation (IOH 1980) for the two longer time series streamflow records, the Kilti and Amen. Dry deposition at this distance from the coast is considered negligible (Keywood *et al.*, 1997) and is typically neglected. Cl_{gw} from the 31 shallow groundwater samples was 2.10 mg/l with a standard deviation of 1.33 mg/l. Cl_{wap} was 0.68 mg/l (standard deviation = 0.32 mg/l); this is the most uncertain parameter of Eqn. 3 in the manuscript given the limited amount of samples as, ideally, samples from throughout the wet season should be obtained. However, the Cl_{wap} value compares well with other studies (Table S6) giving confidence that the value used is representative of rainfall chloride concentrations in this region. The use of few rainfall samples and corroboration with other studies is not uncommon, e.g. Bazuhair and Wood (1996), Subyani (2004), and in South Africa Cl_{wap} is often unmeasured and simply approximated to 1 mg/l (Dennis, 2017), e.g. Butler and Verhagen (2001), and many theses available online.

Table S6. Comparison of rainfall chloride concentrations with other studies

Cl_{wap} (mg/l)	Source	Region	Altitude (m asl)	Distance to coast (km)	Rainfall (mm/a)
0.68	This study	Dangila, NW Ethiopia	~2000	~600	1541
0.50	Kebede *et al.* (2005)	Tana Basin, NW Ethiopia	~2000	~600	~1500
0.86	Asmerom (2008)	Tana Basin, NW Ethiopia	~2000	~600	~1500
0.70	Demlie *et al.* (2007)	Addis Ababa, Central Ethiopia	~2300	~550	1254
0.71±0.18	Vallet-Coulomb *et al.* (2001)	Lake Ziway, Central Ethiopia	~1650	~600	~900

9. Water table fluctuation (WTF) and rainfall infiltration breakthrough (RIB)

The locations of the hand-dug wells used in the WTF and RIB analyses are presented in Fig. S4. Details of the five monitoring wells initially set up as part of the AMGRAF project and the community-based monitoring programme can be found in Walker *et al.* (2016). The depth to groundwater was measured every two days by a community-nominated observer using a dip meter at 6am (prior to well use). The ILSSI project (Innovation Lab for Small-Scale Irrigation funded by USAID) community-based monitoring programme operated in a similar fashion. Two local community members using dip-meters at 6am monitored groundwater level in twenty-five hand-dug wells. Measurements were made weekly in the dry season and daily through the wet season.

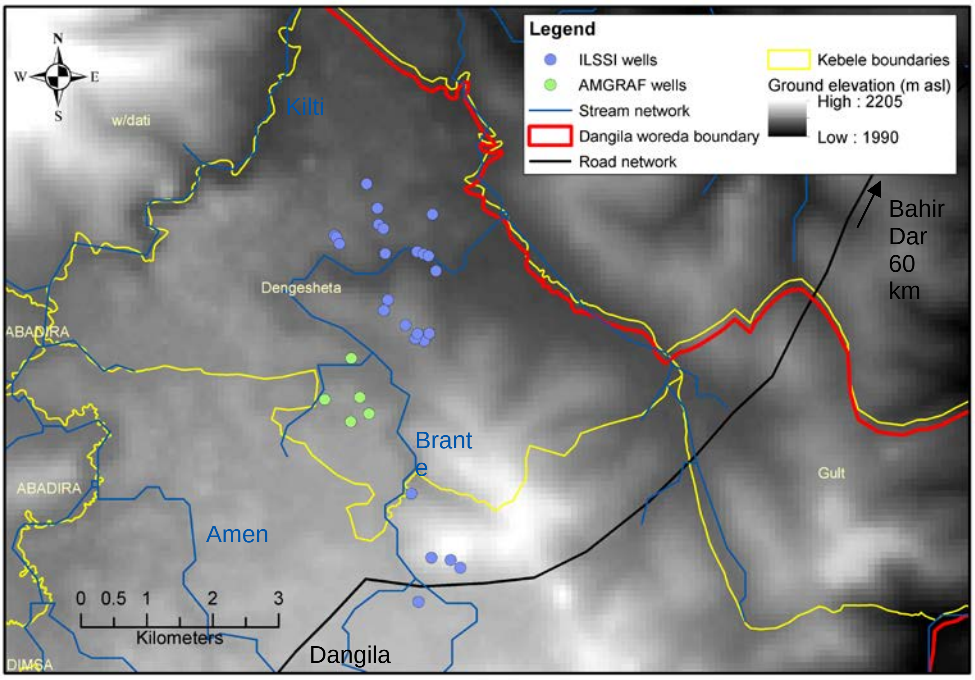

Fig. S4. Location map of the AMGRAF and ILSSI monitoring wells (a *kebele* is similar to a parish).

The WTF method has the key assumption that, because recharge rates vary substantially within a catchment due to differences in elevation, geology, slope, vegetation, and other factors, monitoring wells should be sited so the water levels are representative of the entire catchment (Healy and Cook 2002). Identifying a "representative" location is problematic, therefore, the possibility of recording groundwater level change due to lateral groundwater flow is considerable.

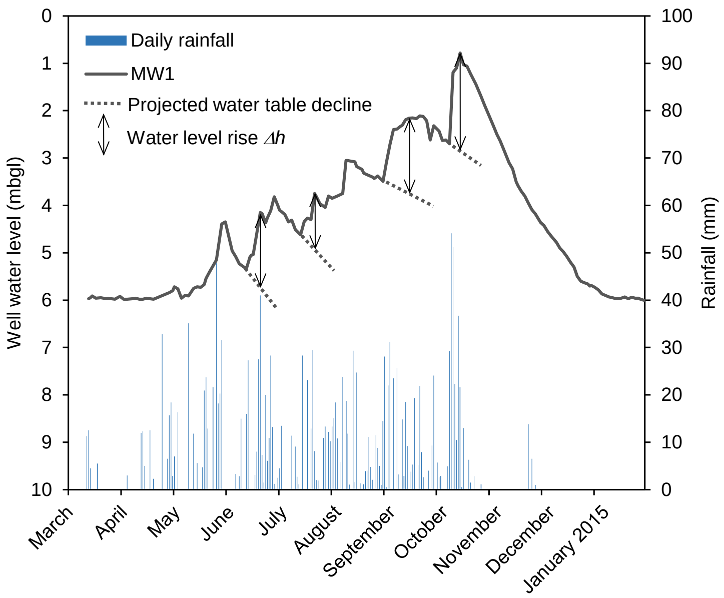

Fig. S5. Groundwater hydrograph through the wet season and determination of water table rise for the WTF method. *MW1* refers to the groundwater level in monitoring well 1 from where this snapshot is taken. (*mbgl* = metres below ground level)

Two RIB method parameters, "*lag days*" and "*length days*", were calibrated within the model. "Lag days" indicates the time it takes percolating rainwater to reach the water table. It was assigned as 1 day (pre and post-calibration) because the shallow water table leads to a short time lag between rainfall and groundwater level peak. "Length days" refers to the length of related rainfall events and is adjusted to gain the best fit between simulated and observed groundwater levels (Fig. S6). Lateral groundwater inflows and outflows can be specified in the model though the difficulty in quantifying these means they are typically set at zero under the assumption that they are in balance. As with the WTF method, this generates the possibility of accounting for groundwater level rise from lateral flows in recharge estimation.

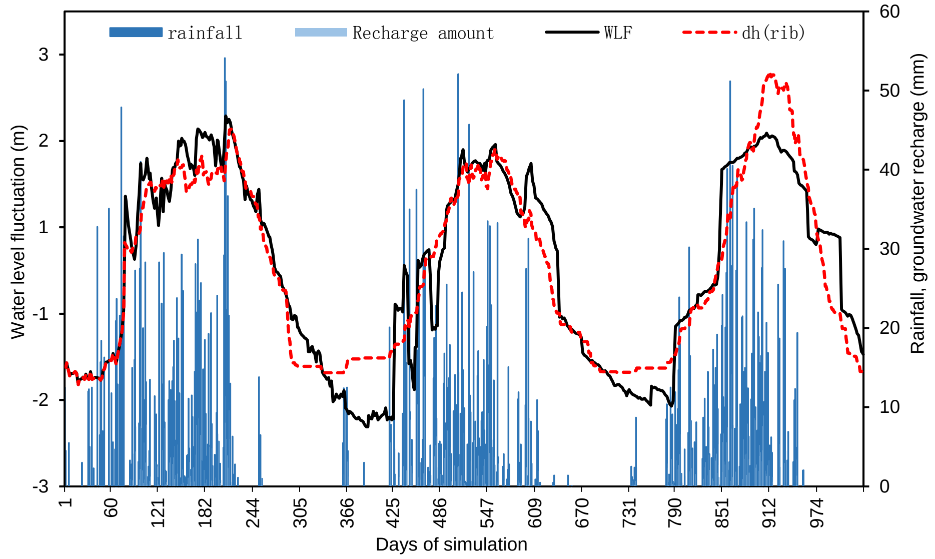

Fig. S6. Graphical output of the RIB model showing observed rainfall, observed groundwater level fluctuation (*WLF*), simulated groundwater level fluctuation (*dh (rib)*) and computed recharge. This plot shows the simulation of monitoring well MW3.

10. SHETRAN modelling

The main advantages of SHETRAN over alternative physically based spatially distributed river basin modelling systems are its comprehensive nature and capabilities for modelling subsurface flow and transport. The subsurface is treated as a variably saturated heterogeneous porous medium, and fully three-dimensional flow and transport can be simulated for combinations of confined, unconfined, and perched systems. The ''unsaturated zone'' is modelled as an integral part of the subsurface, and subsurface flow and transport are coupled directly to surface flow and transport (Ewen *et al.*, 2000). SHETRAN is well established in the literature, having been applied to a variety of situations such as predicting climate and land use change impacts on a Mediterranean catchment (Parkin *et al.*, 1996), modelling landslide sediment yield in Scotland (Burton and Bathurst, 1998), coupling flow and

nitrogen transport in Sweden (Birkinshaw and Ewen, 2000), and modelling forest impact on floods caused by extreme rainfall and snowmelt in Latin America (Bathurst *et al.*, 2011).

Both saturated and unsaturated zones of the subsurface are represented by a single equation:

$$\eta\frac{\partial\psi}{\partial t} = \frac{\partial}{\partial x}[K_x k_r \frac{\partial\psi}{\partial x}] + \frac{\partial}{\partial y}[K_y k_r \frac{\partial\psi}{\partial y}] + \frac{\partial}{\partial z}[K_z k_r \frac{\partial\psi}{\partial z}] + \frac{\partial(k_r K_z)}{\partial z} - q \qquad \text{(E6)}$$

where

$$\eta = \frac{\theta S_s}{n} + \frac{d\theta}{d\psi} \qquad \text{(E7)}$$

and

k_r = relative hydraulic conductivity (-)

(K_x,K_y,K_z) = principal components of saturated hydraulic conductivity (m/s)

n = porosity (-)

q = specific volumetric flow rate out of the porous medium (general source/sink term) (1/s)

S_s = specific storage (1/m)

t = time (s)

(x,y,z) = ordinates of the position vector (m)

θ = volumetric soil water content (-)

η = storage coefficient (1/m)

ψ = pressure potential (m)

As this equation is continuous across saturated-unsaturated boundaries, fluxes across the boundary are implicit in the solution, unlike groundwater models such as MODFLOW where the control volume is the saturated zone defined by the time-varying water table as its upper boundary, and recharge is defined separately as a boundary input. In SHETRAN, therefore, recharge is a derived variable, which includes both the flux (vertical flow per unit area) through the moving water table, and the rate of capture (or loss) of water in the saturated zone as the water table position moves. There is also no explicit use of a variable equivalent to specific yield, so an approximation of this concept is defined here as the amount of water available due to drainage of pore water space above the water table, derived from the shape of the unsaturated zone characteristic functions. Recharge is therefore calculated as:

$$Q_{rch} = -Q_v + S_y \, {dH}/{dt} \qquad \text{(E8)}$$

where

H = phreatic surface (i.e. water table) level (m)

Q_{rch} = recharge rate (m/s)

Q_v = vertical velocity (m/s), +ve upwards

dH = change in phreatic surface level (m), approximated as change in pressure potential ($\Delta\psi$) over the timestep in the highest saturated cell at the end of the timestep

S_y = specific yield (-), approximated as change in water content ($\Delta\theta$) in the cell above the water table over the timestep, implemented as $\eta * \Delta\psi$

dt = timestep (s)

SHETRAN was manually calibrated using an iterative approach with the adjustment of geological layer thicknesses, aquifer properties, channel characteristics, Strickler overland flow roughness coefficient, and evapotranspiration characteristics, which are reported to be the most sensitive parameters (Starkey *et al.*, 2017). The range of values used for the input parameters was determined from field investigations and literature review of models set up for similar climates. Calibration aimed to minimise the error between observed and simulated time series. Calibration and validation periods were selected to give "typical" ranges of hydrological conditions and ran from the end of a wet season recession to the same point one, two or three years later. In addition to visual comparison of plotted observed and simulated data, the following performance indicators were utilised: Nash-Sutcliffe Efficiency (NSE) coefficient (Nash and Sutcliffe, 1970), where a value greater than 0.5 is considered acceptable (Moriasi *et al.*, 2007), and root mean square error (RMSE), with units matching the compared data thus the value should be as low as possible. NSE is very sensitive to peak flows (Krause *et al.*, 2005), therefore, given the flashy nature of the rivers with short-lived and relatively extremely high peaks, NSE (and RMSE) was calculated on low flows following hydrograph separation. For the Brante model, NSE and RMSE were calculated on groundwater levels in the five monitoring wells selected at the onset of the AMGRAF project. The Brante model was principally calibrated against monitored groundwater levels (with consideration of river flow) combined with a semi-quantitative calibration to other areas of the catchment where simulations were compared against occasional observations and anecdotal evidence of frequent flooding or wells prone to drying up. The Amen and Kilti models were chiefly calibrated against river flow, though again with consideration of groundwater level information from around the catchments. A validation period was run to confirm that the calibrated parameters still produced a satisfactory simulation for independent input datasets. Table S7 shows that the calibration statistics are acceptable for both the calibration and validation periods for all catchment models.

Table S7. Details and statistics of the calibration and validation periods for the SHETRAN catchment models

Catchment	Calibration period	No. of days	NSE	RMSE
Amen	5 Apr 1999 to 4 Apr 2001 (years 2-3)	731	0.79	0.19 m^3/s
Kilti	16 Apr 1998 to 15 Apr 2000 (years 2-3)	731	0.78	1.47 m^3/s
Brante	12 Mar 2014 to 11 Mar 2015 (year 1)	365	0.69	2.01 m
	**Validation period**	**No. of days**	**NSE**	**RMSE**
Amen	12 Mar 2010 to 11 Mar 2013 (years 13-15)	1096	0.75	0.13 m^3/s
Kilti	2 Apr 2004 to 2 Apr 2007 (years 8-10)	1096	0.67	2.30 m^3/s
Brante	12 Mar 2015 to 11 Mar 2016 (year 2)	365	0.53	2.08 m

11. Comparison of recharge results from the three nested catchments

In this case, catchment scale means 37-632 km^2 for the three catchments in this study. Despite being nested, the catchments have slightly different characteristics, in terms of proportional land cover and topography, therefore, differences in recharge result would be expected. Additionally, it is useful to evaluate if the different spatial and temporal scales contribute to the discrepancies in recharge results between catchments. The Amen and Kilti analyses utilise 17+ years of input data, the Brante only three, with little overlap. What's more, those three years include 2014; the wettest year on record with annual rainfall of 2005 mm, and 2015 with annual rainfall only in the 20th percentile (1390 mm). Fig. S7 shows the recharge results plotted for each catchment. While no spatial scale dependence can be seen, temporally, the shorter and alternative data periods of the Brante catchment are contributing to the reduced recharge estimate of the streamflow hydrograph methods. For each year from 2014-2017, the streamflow hydrograph methods give much lower recharge estimates than for the longer period analyses of the Amen and Kilti. However, the Brante catchment is flatter with the greatest proportion of floodplain wetlands and shallow water tables; therefore, direct groundwater evaporation would be elevated causing the low *minimum* recharge computations.

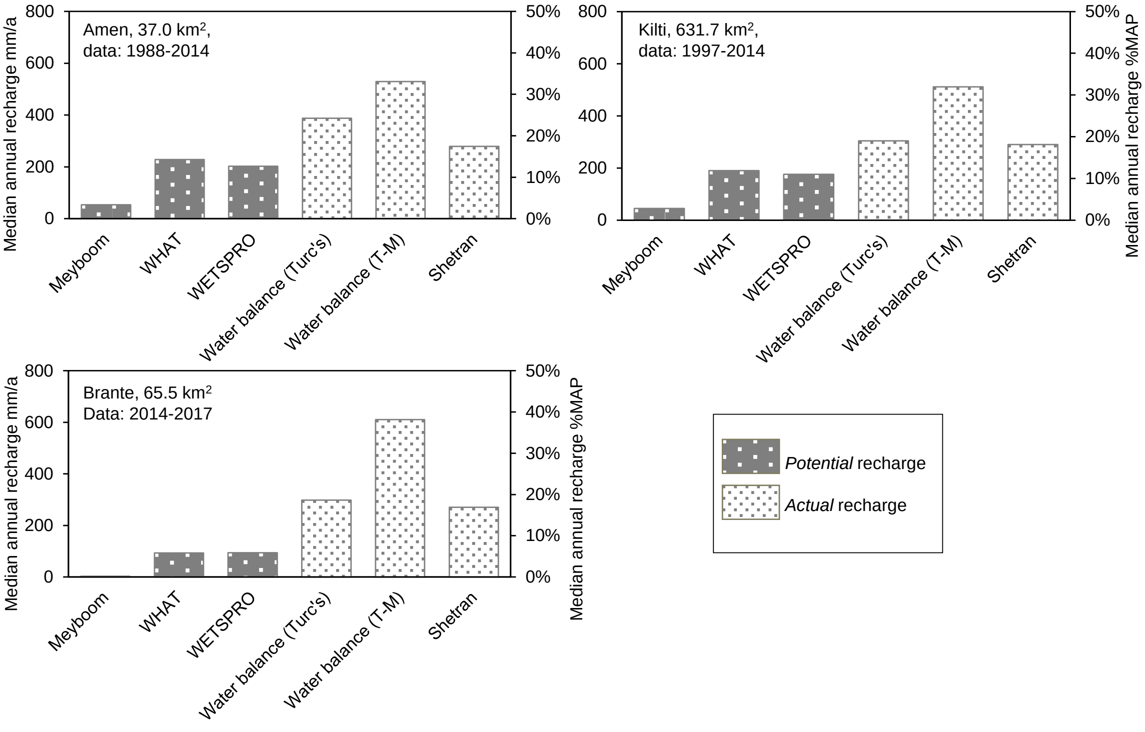

Fig. S7. Graphical comparison of annual recharge estimates from the catchment-scale techniques separated into catchments. *T-M* = Thornthwaite-Mather method of AET estimation.

12. Insights gained on the conceptual model in other recharge studies

Other studies exist where fewer methods were applied and useful insights were gained. These insights are specific to the conceptual model of the study site. King *et al.* (2017) applied four recharge estimation methods to an alluvial aquifer in Queensland, Australia. Low CMB recharge results indicated that inherent assumptions were invalidated and channel leakage and overland flow were significant at the site. The WTF method gave the highest recharge results, especially in proximity to a river, which was considered, since the timing coincided with high stream levels, to be due to temporary influxes of water (bank storage). While a water balance gave a useful approximation of recharge for the catchment, only by applying and comparing the additional methods could the conceptual model be updated and understood. Takounjou *et al.* (2011) compared a hybrid WTF/water balance method with the CMB method for a humid region of Cameroon. The discrepancy in results led the authors to suggest the CMB method was overestimating due to preferential shallow groundwater flow paths; ultimately, they considered the CMB method to be unsuitable for a humid forested environment). Huang *et al.* (2017), by comparing results from a CMB method with groundwater aging and stable isotope analysis, were able to update the conceptual model for a site in northwest China revealing that no recharge had occurred for >2,500 years, and, as such, potential abstraction of the paleaowater would be unsustainable. Misstear *et al.* (2009) compared SMB, WTF, numerical

groundwater modelling and water balance recharge estimation methods for an aquifer in Ireland. The variations in WTF recharge estimates at particular locations, in comparison with more consistent results from other methods, indicated S_Y variations within the aquifer.

13. References (supporting information)

Abdella, A. (2011) *Sustainable groundwater development and management for irrigation in Raya and Kobo Valleys, northern Ethiopia*. MSc thesis, Department of Earth Sciences, Addis Ababa University, Ethiopia.

Abiy, A.Z., Demissie, S.S., MacAlister, C., Dessu, S.B. and Melesse, A.M. (2016) 'Groundwater Recharge and Contribution to the Tana Sub-basin, Upper Blue Nile Basin, Ethiopia. ', in *Landscape Dynamics, Soils and Hydrological Processes in Varied Climates*. Springer, pp. 463-481.

Addisu, D. (2012) *Hydrogeochemical and isotope hydrology in investigating groundwater recharge and flow processes, Lower Afar, Eastern Ethiopia*. MSc thesis, Department of Earth Sciences, Addis Ababa University, Ethiopia.

ADSWE (2015) *Technical Report: Land Use Land Cover and Change Detection, Amhara National Regional State, Bureau of Environmental Protection, Land Administration and Use (BoEPLAU).* . Tana Sub Basin Integrated Land Use Planning and Environmental Impact Study Project, Amhara Design & Supervision Works Enterprise (ADSWE), Bahir Dar, Ethiopia, 71pp.

Alene, Y. (2006) *GIS and remote sensing assisted water balance computation of the Geba basin, Tigray, Ethiopia*. MSc thesis, Water Resources Engineering, KULeuven - VUB, Belgium.

Ali Jr, K.A. (2006) 'Groundwater resources assessment in hard rock terrain using conventional remote sensing and GIS approach in Southern peripheral part of Ethiopia.', *GSA (Geological Society of America) Philadelphia Annual Meeting, 22-25 October 2006*. Philadelphia, USA.

Allam, M.M., Jain Figueroa, A., McLaughlin, D.B. and Eltahir, E.A.B. (2016) 'Estimation of evaporation over the upper Blue Nile basin by combining observations from satellites and river flow gauges', *Water Resources Research*, 52(2), pp. 644-659.

Allen, R.G., Pereira, L.S., Raes, D. and Smith, M. (1998) 'Crop evapotranspiration - Guidelines for computing crop water requirements (FAO Irrigation and Drainage Paper 56)', *Food and Agriculture Organisation of the United Nations, Rome, Italy*.

Altchenko, Y. and Villholth, K.G. (2015) 'Mapping irrigation potential from renewable groundwater in Africa - a quantitative hydrological approach', *Hydrology and Earth System Sciences*, 19(2), pp. 1055-1067.

Andualem, E. (2008) *Evaluation of Ground Water Resources Potential of the Teji River Cathment South West Shoa Zone, Oromia Region*. MSc thesis, School of Earth Sciences, Addis Ababa University, Ethiopia.

Asmerom, G.H. (2008) *Groundwater contribution and recharge estimation in the Upper Blue Nile flows, Ethiopia*. MSc thesis, ITC, Enschede, The Netherlands, 133pp.

Ayalew, H. (2010) *Geological framework for groundwater occurence in Lake Tana basin, northwestern Ethiopia, Amhara region*. MSc thesis, Department of Earth Sciences, Addis Ababa University, Ethiopia.

Aychluhim, D. (2006) *Integrated water resources potential investigation of the Weybo River Catchment Welayita-Hadiya Zones, Southern Ethiopia*. MSc thesis, Department of Earth Sciences, Addis Ababa University, Ethiopia.

Ayenew, T. (2008) 'Hydrological system analysis and groundwater recharge estimation using semi-distributed models and river discharge in the Meki River Basin', *SINET: Ethiopian Journal of Science*, 31(1), pp. 29-42.

Ayenew, T., Demlie, M. and Wohnlich, S. (2008) 'Hydrogeological framework and occurrence of groundwater in the Ethiopian aquifers', *Journal of African Earth Sciences*, 52(3), pp. 97-113.

Ayenew, T., GebreEgziabher, M., Kebede, S. and Mamo, S. (2013) 'Integrated assessment of hydrogeology and water quality for groundwater-based irrigation development in the Raya Valley, northern Ethiopia', *Water International*, 38(4), pp. 480-492.

Ayenew, T. and Tilahun, N. (2008) 'Assessment of lake–groundwater interactions and anthropogenic stresses, using numerical groundwater flow model, for a Rift lake catchment in central Ethiopia', *Lakes & Reservoirs: Research & Management*, 13(4), pp. 325-343.

Azagegn, T., Asrat, A., Ayenew, T. and Kebede, S. (2015) 'Litho-structural control on interbasin groundwater transfer in central Ethiopia', *Journal of African Earth Sciences*, 101, pp. 383-395.

Bakundukize, C., Van Camp, M. and Walraevens, K. (2011) 'Estimation of groundwater recharge in Bugesera region (Burundi) using soil moisture budget approach', *Geologica belgica*, 14(1-2), pp. 85-102.

Bathurst, J.C., Birkinshaw, S.J., Cisneros, F., Fallas, J., Iroume, A., Iturraspe, R., Novillo, M.G., Urciuolo, A., Alvarado, A., Coello, C., Huber, A., Miranda, M., Ramirez, M. and Sarandon, R. (2011) 'Forest impact on floods due to extreme rainfall and snowmelt in four Latin American environments 2: Model analysis', *Journal of Hydrology*, 400(3-4), pp. 292-304.

Bazuhair, A.S. and Wood, W.W. (1996) 'Chloride mass-balance method for estimating ground water recharge in arid areas: examples from western Saudi Arabia', *Journal of Hydrology*, 186(1), pp. 153-159.

BCEOM (1998) *Abbay Basin Master Plan Phase 2 Sectoral Studies, Part 3, Hydrogeology, Main Report*. BCEOM French Engineering Consultants: Ministry of Water Resources (MoWR), Addis Ababa, Ethiopia.

BCEOM (2005) *Harar water supply project groundwater resources*. BCEOM French Engineering Consultants: Ministry of Water Resources (MoWR), Addis Ababa, Ethiopia.

Belay, E.A. (2009) *Growing lake with growing problems: integrated hydrogeological investigation on Lake Beseka, Ethiopia*. PhD thesis, The Faculty of Mathematics and Natural Sciences, University of Bonn, Germany.

Belay, M. and Bewket, W. (2013) 'Traditional irrigation and water management practices in highland Ethiopia: Case study in Dangila woreda', *Irrigation and Drainage*, 62(4), pp. 435-448.

Berehanu, B., Azagegn, T., Ayenew, T. and Masetti, M. (2017) 'Inter-Basin Groundwater Transfer and Multiple Approach Recharge Estimation of the Upper Awash Aquifer System', *Journal of Geoscience and Environment Protection*, 5(03), p. 76.

Berhail, S., Ouerdachi, L. and Keblouti, M. (2015) 'Combination of two evolutionary methods for groundwater recharges estimation in semi-arid regions, Northeastern Algeria', *International Journal of Water*, 9(3), pp. 286-301.

Birkinshaw, S.J. and Ewen, J. (2000) 'Nitrogen transformation component for SHETRAN catchment nitrate transport modelling', *Journal of Hydrology*, 230(1-2), pp. 1-17.

Bonsor, H.C. and MacDonald, A.M. (2010) 'Groundwater and climate change in Africa: review of recharge studies', *British Geological Survey Internal Report*, IR/10/075, p. 30pp.

Burton, A. and Bathurst, J.C. (1998) 'Physically based modelling of shallow landslide sediment yield at a catchment scale', *Environmental Geology*, 35(2-3), pp. 89-99.

Butler, M.J. and Verhagen, B.T. (2001) 'Isotope studies of a thick unsaturated zone in a semi-arid area of Southern Africa', *Isotope Based Assessment of Groundwater Renewal in Water Scarce Regions, IAEA-Tecdoc-1246*, pp. 45-70.

Cain, J.D. (1998) *Modelling evaporation from plant canopies*. Institute of Hydrology Report no. 132, Wallingford, UK, 60pp.

Canadell, J., Jackson, R.B., Ehleringer, J.B., Mooney, H.A., Sala, O.E. and Schulze, E.D. (1996) 'Maximum rooting depth of vegetation types at the global scale', *Oecologia*, 108(4), pp. 583-595.

Chapman, T. (1999) 'A comparison of algorithms for stream flow recession and baseflow separation', *Hydrological Processes*, 13(5), pp. 701-714.

Chishugi, J.B. and Alemaw, B.F. (2009) *World Environmental and Water Resources Congress 2009: Great Rivers*.

Crosbie, R.S., Jolly, I.D., Leaney, F.W. and Petheram, C. (2010) 'Can the dataset of field based recharge estimates in Australia be used to predict recharge in data-poor areas?', *Hydrology and Earth System Sciences*, 14(10), p. 2023.

Dardanelli, J.L., Bachmeier, O.A., Sereno, R. and Gil, R. (1997) 'Rooting depth and soil water extraction patterns of different crops in a silty loam Haplustoll', *Field Crops Research*, 54(1), pp. 29-38.

Demlie, M. (2015) 'Assessment and estimation of groundwater recharge for a catchment located in highland tropical climate in central Ethiopia using catchment soil–water balance (SWB) and chloride mass balance (CMB) techniques', *Environmental Earth Sciences*, 74(2), pp. 1137-1150.

Demlie, M., Wohnlich, S., Gizaw, B. and Stichler, W. (2007) 'Groundwater recharge in the Akaki catchment, central Ethiopia: evidence from environmental isotopes (δ18O, δ2H and 3H) and chloride mass balance', *Hydrological processes*, 21(6), pp. 807-818.

Dennis, R. (2017) 'Regional recharge estimation by applying a genetic algorithm to various groundwater balance methods.', *Presented at the 44th IAH (International Association of Hydrogeologists) Congress, 25th-29th September 2017, Dubrovnik, Croatia*.

Döll, P. and Fiedler, K. (2007) 'Global-scale modeling of groundwater recharge', *Hydrology and Earth System Sciences Discussions*, 4(6), pp. 4069-4124.

Döll, P. and Flörke, M. (2005) 'Global-scale estimation of diffuse groundwater recharge: model tuning to local data for semi-arid and arid regions and assessment of climate change impact', *Frankfurt Hydrology Paper 03, Institute of Physical Geography, Frankfurt University, Frankfurt am Main, Germany*, p. 21pp.

Doorenbos, J. and Pruitt, W.O. (1975) 'Guidelines for predicting crop water requirements (FAO Irrigation and Drainage Paper 24)', *Food and Agriculture Organisation of the United Nations, Rome, Italy*.

Eckhardt, K. (2005) 'How to construct recursive digital filters for baseflow separation', *Hydrological processes*, 19(2), pp. 507-515.

Eckhardt, K. (2008) 'A comparison of baseflow indices, which were calculated with seven different baseflow separation methods', *Journal of Hydrology*, 352(1), pp. 168-173.

Enku, T., Melesse, A.M., Ayana, E.K., Tilahun, S.A., Abate, M. and Steenhuis, T.S. (2016) 'Groundwater-Evaporation and Recharge for a floodplain in a Sub-Humid Monsoon Climate in Ethiopia', *Land Degradation & Development*.

Ewen, J., Parkin, G. and O'Connell, P.E. (2000) 'Shetran: distributed river basin flow and transport modeling system', *Journal of Hydrologic Engineering*, 5(3), pp. 250-258.

Fan, J., McConkey, B., Wang, H. and Janzen, H. (2016) 'Root distribution by depth for temperate agricultural crops', *Field Crops Research*, 189, pp. 68-74.

Furi, W., Razack, M., Haile, T., Abiye, T.A. and Legesse, D. (2011) 'The hydrogeology of Adama-Wonji basin and assessment of groundwater level changes in Wonji wetland, Main Ethiopian Rift: results from 2D tomography and electrical sounding methods', *Environmental Earth Sciences*, 62(6), pp. 1323-1335.

Galloway, D.L. and Hoffmann, J. (2007) 'The application of satellite differential SAR interferometry-derived ground displacements in hydrogeology', *Hydrogeology Journal*, 15(1), pp. 133-154.

Galloway, D.L., Hudnut, K.W., Ingebritsen, S.E., Phillips, S.P., Peltzer, G., Rogez, F. and Rosen, P.A. (1998) 'Detection of aquifer system compaction and land subsidence using interferometric synthetic aperture radar, Antelope Valley, Mojave Desert, California', *Water Resources Research*, 34(10), pp. 2573-2585.

Gebremeskel, G. (2015) *Estimation of groundwater recharge and potentials under changing climate in Werii Watershed, Tekeze River Basin*. MSc thesis, School of Natural Resource and Environmental Engineering, Haramaya University, Ethiopia.

Gebreyohannes, T., De Smedt, F., Walraevens, K., Gebresilassie, S., Hussien, A., Hagos, M., Amare, K., Deckers, J. and Gebrehiwot, K. (2013) 'Application of a spatially distributed water balance model for assessing surface water and groundwater resources in the Geba basin, Tigray, Ethiopia', *Journal of Hydrology*, 499, pp. 110-123.

Gibb and Seureca (1996) *The town of Harar water supply, Hydrogeological study interim working paper*. Sir Alexander Gibb and Seureca Consulting Engineers: Ministry of Water Resources (MoWR), Addis Ababa, Ethiopia.

Girmay, E., Ayenew, T., Kebede, S., Alene, M., Wohnlich, S. and Wisotzky, F. (2015) 'Conceptual groundwater flow model of the Mekelle Paleozoic–Mesozoic sedimentary outlier and surroundings (northern Ethiopia) using environmental isotopes and dissolved ions', *Hydrogeology Journal*, 23(4), pp. 649-672.

Gooddy, D.C., Darling, W.G., MacDonald, A.M. and Morris, B.L. (2012) 'The practicalities of using CFCs and SF6 for groundwater dating and tracing', *Applied Geochemistry*, 27(9), pp. 1688-1697.

Greitzer, Y. (1970) *Stratigraphy, hydrogeology and Jurassic ammonites of the Harar and Diredawa area, Ethiopia*. PhD thesis, Hebrew University, Jerusalem, Israel.

Habtamu, G.D. (2009) *Groundwater Potential Investigation of upper wabe river catchment, south eastern central Ethiopia*. MSc thesis, Department of Earth Sciences, Addis Ababa University, Ethiopia.

Hagos, M.A. (2010) *Groundwater Flow Modelling assisted by GIS and RS Techniques (Raya Valley-Ethiopia)*. MSc thesis, ITC, Enschede, The Netherlands.

Healy, R.W. (2010) *Estimating groundwater recharge*. Cambridge University Press, Cambridge, UK, 256pp.

Huang, T., Pang, Z., Liu, J., Yin, L. and Edmunds, W.M. (2017) 'Groundwater recharge in an arid grassland as indicated by soil chloride profile and multiple tracers', *Hydrological Processes*, 31(5), pp. 1047-1057.

Hussen, E. (2006) *Water resource potential evaluation of Berga River Catchment, West Shewa Zone, Oromiya Regional State*. MSc thesis, Department of Earth Sciences, Addis Ababa University, Ethiopia.

IOH (1980) *Low Flow Studies*. Institute of Hydrology, Wallingford, UK.

Kahsay, G.H. (2008) *Groundwater resource assessment through distributed steady-state flow modeling, Aynalem wellfield (Mekele, Ethiopia)*. MSc thesis, ITC, Enschede, The Netherlands.

Kebede, S., Travi, Y., Alemayehu, T. and Ayenew, T. (2005) 'Groundwater recharge, circulation and geochemical evolution in the source region of the Blue Nile River, Ethiopia', *Applied Geochemistry*, 20(9), pp. 1658-1676.

Keywood, M.D., Chivas, A.R., Fifield, L.K., Cresswell, R.G. and Ayers, G.P. (1997) 'The accession of chloride to the western half of the Australian continent', *Soil Research*, 35(5), pp. 1177-1190.

King, A.C., Raiber, M., Cox, M.E. and Cendón, D.I. (2017) 'Comparison of groundwater recharge estimation techniques in an alluvial aquifer system with an intermittent/ephemeral stream (Queensland, Australia)', *Hydrogeology Journal*, pp. 1-19.

Koch, F.J., van Griensven, A., Uhlenbrook, S., Tekleab, S. and Teferi, E. (2012) 'The Effects of land use change on hydrological responses in the choke mountain range (Ethiopia) - a new approach addressing land use dynamics in the model SWAT', *Proceedings of 2012 international congress on environmental modeling and software managing resources of a limited planet, sixth biennial meeting, Leipzig, Germany*. Citeseer, pp. 1-5.

Krause, P., Boyle, D.P. and Bäse, F. (2005) 'Comparison of different efficiency criteria for hydrological model assessment', *Advances in Geosciences*, 5, pp. 89-97.

Kumai, H. and Mitamura, M. (2004) 'Application of stream hydrograph separation method to estimate the recharge in the northern part of the Bandung Basin, west Java, Indonesia', *Journal of Groundwater Hydrology*, 46(3), pp. 213-225.

Lim, K.J., Engel, B.A., Tang, Z., Choi, J., Kim, K.S., Muthukrishnan, S. and Tripathy, D. (2005) 'Automated web GIS based hydrograph analysis tool, WHAT', *JAWRA Journal of the American Water Resources Association*, 41(6), pp. 1407-1416.

L'vovich, M.I. (1979) *World water resources and their future*. American Geophysical Union, Washington DC, USA, 264pp.

MacDonald, A.M., Bonsor, H.C., Dochartaigh, B.E.O. and Taylor, R.G. (2012) 'Quantitative maps of groundwater resources in Africa', *Environmental Research Letters*, 7(2), p. 7.

Mau, D.P. and Winter, T.C. (1997) 'Estimating ground-water recharge from streamflow hydrographs for a small mountain watershed in a temperate humid climate, New Hampshire, USA', *Groundwater*, 35(2), pp. 291-304.

Mechal, A., Wagner, T. and Birk, S. (2015) 'Recharge variability and sensitivity to climate: The example of Gidabo River Basin, Main Ethiopian Rift', *Journal of Hydrology: Regional Studies*, 4, pp. 644-660.

Meyboom, P. (1961) 'Estimating ground-water recharge from stream hydrographs', *Journal of Geophysical Research*, 66(4), pp. 1203-1214.

Misstear, B.D.R., Brown, L. and Johnston, P.M. (2009) 'Estimation of groundwater recharge in a major sand and gravel aquifer in Ireland using multiple approaches', *Hydrogeology Journal*, 17(3), pp. 693-706.

Moriasi, D.N., Arnold, J.G., Van Liew, M.W., Bingner, R.L., Harmel, R.D. and Veith, T.L. (2007) 'Model evaluation guidelines for systematic quantification of accuracy in watershed simulations', *Transactions of the ASABE*, 50(3), pp. 885-900.

NASA (2016) 'Amazing GRACE https://science.nasa.gov/science-news/science-at-nasa/2001/ast30oct_1', *Accessed 21 December 16*.

Nash, J.E. and Sutcliffe, J.V. (1970) 'River flow forecasting through conceptual models part I - A discussion of principles', *Journal of Hydrology*, 10(3), pp. 282-290.

Nedaw, D. (2010) 'Water Balance and Groundwater Quality of Koraro Area, Tigray, Northern Ethiopia', *Momona Ethiopian Journal of Science*, 2(2), pp. 110-127.

Netsanet, K. (2007) *Groundwater Resources Evaluation and Management in Dugda Woreda, Central Rift Valley, Ethiopia*. MSc thesis, Department of Earth Sciences, Addis Ababa University, Ethiopia.

Nuramo, D. (2016) *Temporal changes in Groundwater Recharge in the Upper Awash Basin with particular emphasis to Becho and Koka areas, Central Ethiopia*. MSc thesis, Department of Earth Sciences, Addis Ababa University, Ethiopia.

Parkin, G., Odonnell, G., Ewen, J., Bathurst, J.C., Oconnell, P.E. and Lavabre, J. (1996) 'Validation of catchment models for predicting land-use and climate change impacts .2. Case study for a Mediterranean catchment', *Journal of Hydrology*, 175(1-4), pp. 595-613.

Reys, A. (2016) *Ground Water Potential Evaluation and Use Trends in Upper Awash Basin: with Special Emphasis to Koka-Becho area*. MSc thesis, Department of Earth Sciences, Addis Ababa University, Ethiopia.

Shawul, A.A., Alamirew, T. and Dinka, M.O. (2013) 'Calibration and validation of SWAT model and estimation of water balance components of Shaya mountainous watershed, Southeastern Ethiopia', *Hydrology and Earth System Sciences Discussions*, 10(11), pp. 13955-13978.

Shimelis, A., Megerssa, O. and Fantahun, A. (2014) 'Estimation of Groundwater Recharge Using Water Balance Model Coupled with Base flow Separation in Bulbul River Catchment of Gilgel-Gibe River Basin, Ethiopia', *Asian Journal Of Applied Science And Engineering*, 3(2), pp. 235-243.

Sintayehu, L.G. (2009) *Integrated Hydrogeological Investigation Of Upper Bilate River Catchment, South Western escarpment of Main Ethiopian Rift*. MSc thesis, Department of Earth Sciences, Addis Ababa University, Ethiopia.

Starkey, E., Parkin, G., Birkinshaw, S., Large, A., Quinn, P. and Gibson, C. (2017) 'Demonstrating the value of community-based ('citizen science') observations for catchment modelling and characterisation', *Journal of Hydrology*, 548(Supplement C), pp. 801-817.

Subyani, A.M. (2004) 'Use of chloride-mass balance and environmental isotopes for evaluation of groundwater recharge in the alluvial aquifer, Wadi Tharad, western Saudi Arabia', *Environmental Geology*, 46(6-7), pp. 741-749.

Tadesse, N., Tadios, S. and Tesfaye, M. (2010) 'The Water Balance of May Nugus Catchment, Tigray, Northern Ethiopia', *International Journal of Earth Sciences and Engineering*, 3(5), pp. 609-625.

Takounjou, A.F., Ngoupayou, J.R.N., Riotte, J., Takem, G.E., Mafany, G., Marechal, J.C. and Ekodeck, G.E. (2011) 'Estimation of groundwater recharge of shallow aquifer on humid environment in Yaounde, Cameroon using hybrid water-fluctuation and hydrochemistry methods', *Environmental Earth Sciences*, 64(1), pp. 107-118.

Tapley, B.D., Bettadpur, S., Ries, J.C., Thompson, P.F. and Watkins, M.M. (2004) 'GRACE measurements of mass variability in the Earth system', *Science*, 305(5683), pp. 503-505.

Teklebirhan, A., Dessie, N. and Tesfamichael, G. (2012) 'Groundwater recharge, evapotranspiration and surface runoff estimation using WetSpass modeling method in Illala catchment, northern Ethiopia', *Momona Ethiopian Journal of Science*, 4(2), pp. 96-110.

Tesfagiorgis, K., Gebreyohannes, T., De Smedt, F., Moeyersons, J., Hagos, M., Nyssen, J. and Deckers, J. (2011) 'Evaluation of groundwater resources in the Geba basin, Ethiopia', *Bulletin of Engineering Geology and the Environment*, 70(3), pp. 461-466.

Tesfaye, T.G. (2010) *Groundwater potential evaluation based on integrated GIS and remote sensing techniques in Bilate River Catchment: South Rift Valley of Ethiopia*. MSc thesis, Department of Earth Sciences, Addis Ababa University, Ethiopia.

Thornthwaite, C.W. and Mather, J.R. (1955) 'The Water Balance', *Publications in climatology*, 8(1), pp. 1-104, Drexel Institute of Climatology, Centerton, New Jersey.

Thornthwaite, C.W. and Mather, J.R. (1957) 'Instructions and tables for computing potential evapotranspiration and the water balance', *Publications in Climatology*, X(3), p. 311.

Tilahun, K. and Merkel, B.J. (2009) 'Estimation of groundwater recharge using a GIS-based distributed water balance model in Dire Dawa, Ethiopia', *Hydrogeology Journal*, 17(6), pp. 1443-1457.

Turc, L. (1954) 'Le bilan d'eau des sols: Relations entre les precipitations, l'evaporation et l'ecoulement', *Annals Agronomiques*, (5), pp. 491-595.

Vallet-Coulomb, C., Legesse, D., Gasse, F., Travi, Y. and Chernet, T. (2001) 'Lake evaporation estimates in tropical Africa (Lake Ziway, Ethiopia)', *Journal of Hydrology*, 245(1–4), pp. 1-18.

van Beek, L.P.H., Wada, Y. and Bierkens, M.F.P. (2011) 'Global monthly water stress: 1. Water balance and water availability', *Water Resources Research*, 47, p. 25.

Vandecasteele, I., Nyssen, J., Clymans, W., Moeyersons, J., Martens, K., Van Camp, M., Gebreyohannes, T., Desmedt, F., Deckers, J. and Walraevens, K. (2011) 'Hydrogeology and groundwater flow in a basalt-capped Mesozoic sedimentary series of the Ethiopian highlands', *Hydrogeology journal*, 19(3), pp. 641-650.

Walker, D., Forsythe, N., Parkin, G. and Gowing, J. (2016) 'Filling the observational void: Scientific value and quantitative validation of hydrometeorological data from a community-based monitoring programme', *Journal of Hydrology*, 538, pp. 713-725.

Walraevens, K., Gebreyohannes Tewolde, T., Amare, K., Hussein, A., Berhane, G., Baert, R., Ronsse, S., Kebede, S., Van Hulle, L. and Deckers, J. (2015) 'Water Balance Components for Sustainability Assessment of Groundwater-Dependent Agriculture: Example of the Mendae Plain (Tigray, Ethiopia)', *Land Degradation & Development*, 26(7), pp. 725-736.

Walraevens, K., Vandecasteele, I., Martens, K., Nyssen, J., Moeyersons, J., Gebreyohannes, T., De Smedt, F., Poesen, J., Deckers, J. and Van Camp, M. (2009) 'Groundwater recharge and flow in a small mountain catchment in northern Ethiopia', *Hydrological Sciences Journal*, 54(4), pp. 739-753.

WHYMAP (2016) 'www.whymap.org World-wide Hydrogeological Mapping and Assessment Programme', *BGR and UNESCO.* , Accessed 21 December 2016.

Willems, P. (2009) 'A time series tool to support the multi-criteria performance evaluation of rainfall-runoff models', *Environmental Modelling & Software*, 24(3), pp. 311-321.

Woldearegay, K. (2004) 'A study of water resource potential of Aba’ala Wereda', *Research and development experience on dryland husbandry in Ethiopia. OSSREA and Mekelle University, Addis Ababa, Ethiopia*, pp. 93-107.

Yihdego, S. (2003) *Hydrogeology of Illala-Aynalem catchments with particular reference to the chemical variation and aquifer characterization*. MSc thesis, Department of Earth Sciences, Addis Ababa University, Ethiopia.

Zeru (2008) *Evaluation of groundwater potential using modeling (MODFLOW)*. MSc thesis, Mekelle University, Ethiopia.

Zewdie, G. (2010) *Baseflow analysis of Rivers in Lake Tana sub basin*. MSc thesis, Department of Earth Sciences, Addis Ababa University, Ethiopia.

Zhang, L., Walker, G.R. and Dawes, W. (1999) 'Predicting the effect of vegetation changes on catchment average water balance', *Cooperative Research Centre for Catchment Hydrology, CSIRO Land and Water, Canberra, Australia.*
